# Effect of small molecule signaling in PepFect14 transfection

**DOI:** 10.1371/journal.pone.0228189

**Published:** 2020-01-30

**Authors:** Maxime Gestin, Henrik Helmfors, Luca Falato, Nicola Lorenzon, Filip Ilias Michalakis, Ülo Langel

**Affiliations:** 1 Department of Biochemistry and Biophysics, Stockholm University, Stockholm, Sweden; 2 Laboratory of Molecular Biotechnology, Institute of Technology, University of Tartu, Nooruse, Tartu, Estonia; Consejo Superior de Investigaciones Cientificas, SPAIN

## Abstract

Cell-penetrating peptides can be used to deliver oligonucleotide-based cargoes into cells. Previous studies have shown that the use of small molecule drugs could be an efficient method to increase the efficacy of delivery of oligonucleotides by cell-penetrating peptides either as targeting agents that can be used in formulation with the cell-penetrating peptide and its cargo or as cell signaling modulators that facilitates the cellular uptake of the treatment. This study presents two aims. The first aim is the identification of small molecule drugs that would induce a synergic effect on the transfection of splice correcting oligonucleotides assisted by PepFect14. The second aim is to identify the mechanisms behind the effect of small molecule drugs modulation of cell-penetrating peptide assisted transfection of oligonucleotides. Through an optimized, high-throughput luciferase assay for short oligonucleotide delivery using cell-penetrating peptides, and the simultaneous addition of a small molecule drug library, we show that three small molecule drugs (MPEP, VU0357121 and Ciproxifan) induced an increase in the transfection efficacy of PepFect14 in complex with a short single-stranded oligonucleotide in HeLa pLuc705 cells. These three drugs are described in the literature to be highly specific for their respective target receptors. However, none of those receptors are expressed in our cell line, indicating a yet non-described pathway of action for these small molecules. We show that the indicated small molecules, without interfering with the particles formed by PepFect14 and the oligonucleotide, interfere via still unidentified interactions in cell signaling, leading to an up-regulation of endocytosis and a higher efficacy in the delivery of short splice correcting oligonucleotides in complex with PepFect14.

## Introduction

Oligonucleotides (ONs) -based therapies have considerable promises as treatments for a variety of different diseases, they could be used to replace a lost gene or gene function [1]; to silence a malfunctioning protein via the RNA interference pathway [2,3] or alter the splicing of an mRNA using splice-correcting oligonucleotides (SCOs) [4]. The reason why ONs have not yet realized their full therapeutic potential is that, unmodified and non-protected, ONs are rapidly degraded in biological fluids, they are too large, too charged and much too hydrophilic to passively enter cells hence violating all of the five properties that make a molecule “*drug like*” [5]. Furthermore, they do not spontaneously accumulate in target tissues. Much research has gone into developing delivery systems for ON therapies including cell-penetrating peptides (CPPs). CPPs have the innate ability to cross cellular membranes and transport various cargoes. Many different research groups have successfully delivered siRNA [6,7], anti-sense oligonucleotides[8,9] and plasmid DNA using CPPs [10,11]. Besides CPPs offer several possibility for achieving a targeted delivery in vivo as described in a review by Kurrikoff et al [12].

One simple way to determine the efficacy of delivery is the use of SCOs in the splice-correcting assay developed by Kang et al [13]. In this assay luciferase is incorrectly spliced, rendering it non-functional, and the successful delivery of SCOs leads to the masking of a cryptic splicing site and the correct expression is restored. Using the CPP PepFect 14 (PF14) to deliver an SCO gives up to a 100-fold increase in luciferase expression splice-correction compared to untreated cells [14]. This assay was recently modified by our group in order to allow the use of plates with more and smaller wells to increase the throughput [15].

PepFect 14 is an amphipathic CPP, composed of a sequence of twenty-one amino acids and a stearic acid covalently bonded at its N-terminus ((Stearyl- AGYLLGKLLOOLAAAALOOLL-NH2). PF14 forms complexes with oligonucleotides and is able to deliver them inside various cell types [16,17]. Its amphipathic structure with cationic residues allows PF14 to bind to negatively charged oligonucleotides and to form self-assembled nanoparticles. PF14 allows the non-covalent binding and the delivery of various types of oligonucleotide such as double stranded plasmid DNA [16], siRNA[18], anti-miRNA[19] and SCO[14]. Self-assembling nanoparticle is a widely used strategy in the field of gene delivery [20–22] as it gives several advantages. First, the condensation of genetic material in a nanoparticle allows a protection against nucleases that would degrade oligonucleotides. Second, it offers a relative rigidity of the structure that protects the complexes from dissociating when in contact with other ions. [23]

It is known that, at least to some extent, cell-surface receptors of the scavenger receptor type A-family (SCARA) are involved in the uptake of both CPP:ON complexes [24], Polymer:ON complexes [25] and naked modified ONs in nanoparticle form [26]. The chemokine receptor type 4 is also known to be involved in the uptake of the CPP oligoarginine, by stimulating macropinocytosis [27]. Furthermore, the neurophilin receptor is reported to internalize cationic peptides that have a K/RXXR/K C-terminal motif [28]. In one of our recent studies [29], we also demonstrated the involvement of autophagy signaling pathways in the uptake of PF14:ON and more particularly a differential regulation of several cell-surface receptors were found when the cells were treated with our complex. Among those receptors can be found connective tissue growth factor (CTGF), low-density lipoprotein receptor (LDLR) or β-adrenergic receptor 2 (ADRB2). We have also shown that the use of agonists or antagonists to these receptors could modulate the efficiency of the delivery of SCOs in complex with PepFect14. [29]

To find out new receptors involvement in the uptake of complexes or nanoparticles, the use of ligand interfering is widely used. The principle is to incubate cells with a drug that modulates a specific protein and to measure the efficiency of delivery depending on the concentration of the ligands. If a dose dependent effect is seen, it suggests that the target receptor or the downstream signal connected to this receptor is of importance in the uptake mechanism of the particle. The use of microplates for such studies is of interest as it allows for large screening of small drugs. An example of ligand library screening is given by Tam et al [30]. They discovered the effect of cardiac glycosides on the transfection of lipid nanoparticles in HeLa cells when using an eight hundred ligands library. After this discovery, they used cardiac glycosides to decorate lipid nanoparticle and they obtained a 3-fold increase of the uptake efficiency.

Despite this example [30] where the use of ligands provides an enhanced uptake for sixteen different cell lines from different origins, ligands are mostly used for achieving a targeted delivery. A cell penetrating nanoparticle is decorated with ligands that present a high affinity for a receptor specific of the cell line or tissue to be treated. A high specificity for a treatment can then be achieved. This targeting technique has been widely used in the recent years as shown by Mészáros et al [31], Song et al [32]. Briefly, in Mészáros et al, niosomes were decorated with glutathione, alanine and glucopyranose to target blood brain barrier (BBB) transporters. This design of the particle induced a 2-fold increase in the penetration through the BBB [31]. Song et al demonstrated the efficiency of three different ligands to increase the transcytosis through biological barriers by targeting the Neonatal Fc receptor, the transferrin receptor and the αvβ3 integrin [32]. These last two studies present the use of ligands as a targeting platform to increase the uptake of nanoparticles in a specific tissue. However ligands can be used as a broad library to discover new interactions occurring during the uptake of delivery platforms [30].

Here we report a high-throughput screening aiming to find new co-treatment of CPP:ON complexes with small molecule drugs giving a modulation of the delivery efficacy and to discover new signaling pathways involved in the transfection of HeLa pLuc705 cells. We obtained a small-molecule drugs library, a mix of allosteric modulators, agonists, antagonists and inverse agonists with diverse targets, consisting of 264 members (S1 Table) and used this library in conjunction with our recently modified splice-correction assay [15] to identify molecules that affect the delivery efficacy of PF14:SCO complexes. The first goal of this study was to identify new cell surface receptors that would be involved in the recognition and uptake of our complexes. We identified three drugs that induced a significant increase in the delivery efficacy. However, the target receptors of these drugs were not found to be expressed in our cell line. Thus, our goal moved to identify the action of the drugs that led to the increase of transfection efficacy. We showed that these drugs enhanced the endocytosis of PF14:SCO through a still unidentified signaling pathway.

## Results and discussion

### Small molecule drugs influence the transfection

The library screening assay was performed on HeLa pLuc705 cells with 100 μM concentration of each drug together with the complexes PF14:SCO. As the drugs were originally dissolved in DMSO, the final DMSO concentration in the wells was 1%. The luminescence resulting from the splice correction was evaluated compared to controls treated only with PF14:SCO in 1% DMSO. A result was deemed to be of interest if it diverged more than 3 times standard deviations from the mean value of the controls, in three independent replicates of the library screening. The ligands that met the criteria in all three replicates were selected for further investigation by dose-titration experiments.

**Fig 1:**
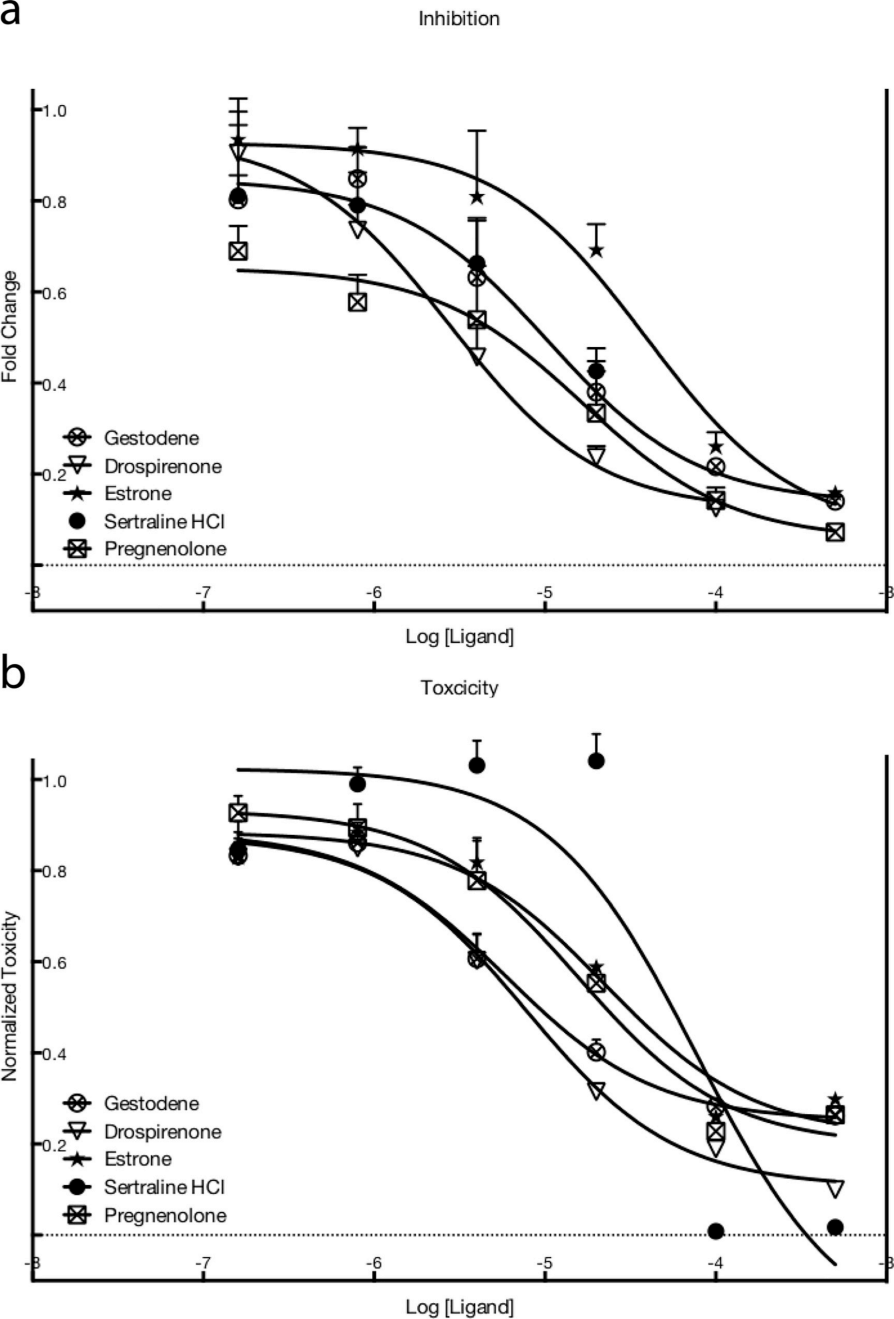
(a) Decrease in luminescence from luciferase activity of HeLa pLuc705 cells after treatment with PF14:SCO together with the drugs, compared as a fold change to control. (b) Toxicity as measured by WST-1 compared to controls. Controls in both a) and b) mean not treated with any ligand. In both A and B the values are expressed as mean values + SEM of four independent experiments.

Five ligands were selected for their inhibitory activity and all were found to dose-dependently decrease the luciferase activity (Fig 1A). They all are estrogen receptor ligands. As it can be seen from Fig 1B, the toxicity effects determined by the WST-1 assay are significant for the ligands that inhibit the luciferase signal. The inhibitory effect given by the five ligands on luciferase luminescence and their effect on cell viability are compared in Table 1. Unfortunately, the inhibitory effects were indistinguishable from toxicity. Comparing the IC50 values for the inhibitory and toxic effects of the five ligands used here showed that while the values are different, they were very similar in size and range, indicating that the discrimination between a possible inhibitory effect and toxic effects is not possible (Table 1). The observed decreased in the luminescence showed a decrease in the amount of the spliced luciferase that is produced. This decrease could be due to either a lower efficiency of the splice correction but also to a decreased cell proliferation. Indeed, a lower amount of cells would induce a lower overall production of luciferase. In our case the inherent toxicity of the drugs treatment decreased the proliferation rate of the cells and the lower amount of cells yielded in an observed decreased production of spliced luciferase. To ensure that the observed toxicity was not due to a combined effect of the drugs and the PF14:SCO complexes, the cell proliferation was also measured when the cells were treated with drugs alone (S1 File). The IC50 of the toxic effect induced by the drugs alone was in the same range than with the PF14:SCO complex except for sertraline HCl that presented a 2.7 times higher IC50 and estrone that presented a 15 fold increased IC50. This result might indicate a combined effect of sertraline and estrone with PF14:SCO. This effect could be due to an interaction between the nanoparticle and the drug but as these molecules were excluded from the rest of the study, these possible interactions is not discussed here.

**Table 1:** Comparison between the toxic effect of the drugs and the apparent inhibition of the luciferase activity induced by the splice correction. Values are calculated from four independent experiments and reported as mean ± SEM. Values are obtained from treatment on HeLa pLuc705.

INHIBITION	Toxicity IC50 μM ± SEM	Inhibition IC50 μM ± SEM
**Gestodene**	0,68 ± 0,14	1,14 ± 0,54
**Drospirenone**	0,70 ± 0,12	0,42 ± 0,13
**Estrone**	2,80 ± 0,28	4,04 ± 1,35
**Sertraline HCl**	4,30 ± 0,25	1,55 ± 0,77
**Pregnenolone**	2,18 ± 0,51	2,69 ± 1,14

These results raise interesting questions about the cause of the toxicity. HeLa cells were originally harvested from a carcinoma in the cervix, so it is not unlikely that these cells express estrogen receptors. The Human Protein Atlas [33] has analyzed the expression of the estrogen receptor 1 in HeLa cells, but depending on which antibody was used, was positive in either less then 10% or more than 79% of the analyzed cells. Rago et al [34] showed a blot with no signal for estrogen receptors in HeLa cells whereas Monje et al [35] presented a blot with an ERβ stain for HeLa cells but no signal for Erα.

**Fig 2:**
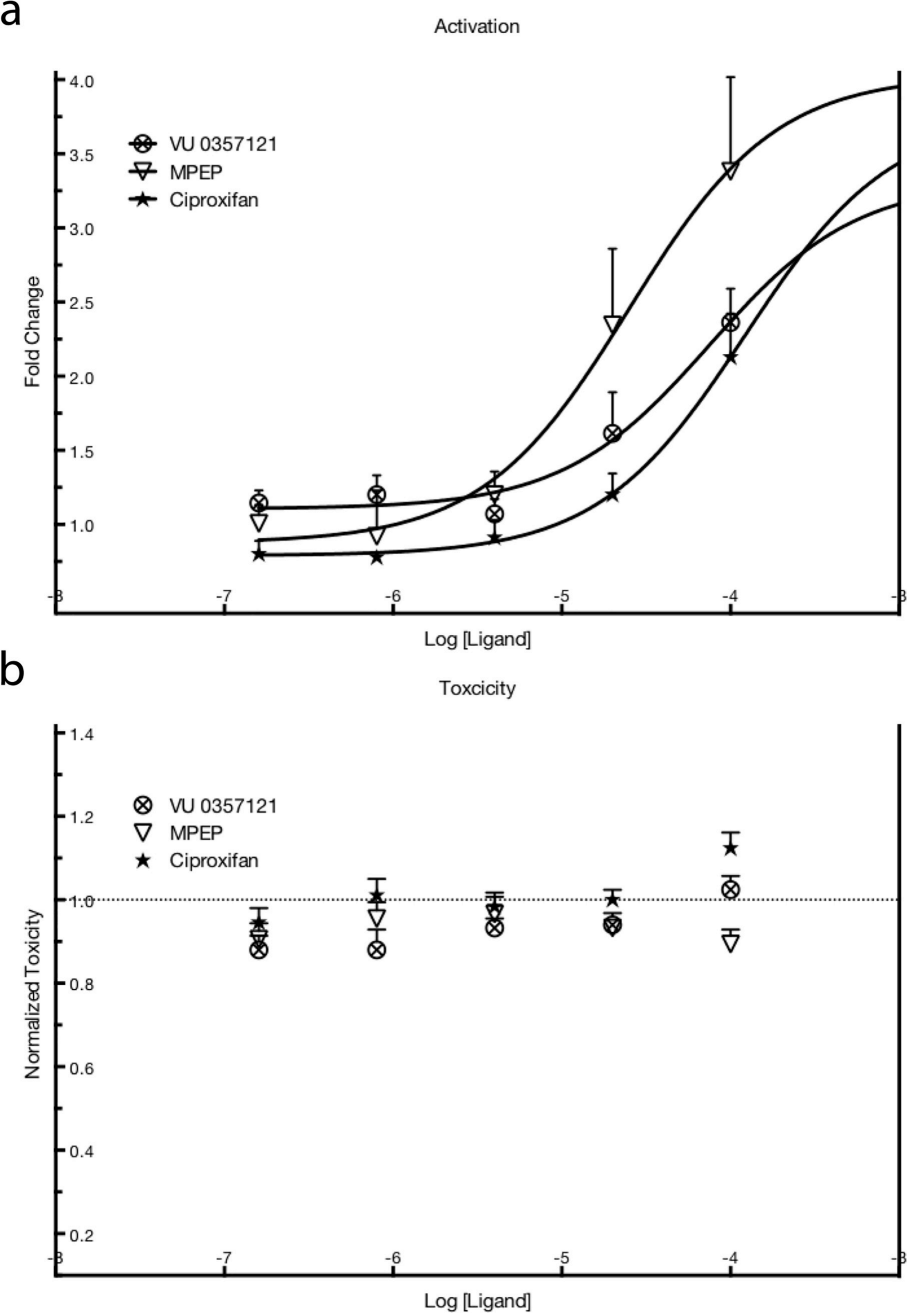
(a) Increase as a fold change in luminescence from luciferase activity after treatment of HeLa pLuc 705 cells with PF14:SCO together with the drugs, compared to the control. (b) Toxicity as measured by WST-1 compared to controls. Controls in both A and B mean not treated with any ligand. In both A and B the values are expressed as mean values + SEM of four independent experiments.

Three ligands were confirmed to have a dose-dependent effect, with over a 3-fold increase in luminescence (Fig 2A). Two of them are known to be allosteric modulators of metabotropic glutamate receptor 5 (MPEP and VU 0357121) and one is a histamine receptor H3 antagonist (ciproxifan). The chemical structures of these three drugs are displayed in S1 Fig. The calculated EC50 values are presented in Table 2, the reported EC/IC50 values for each of the activators to their respective receptor are in the last column of the same table. The three hits from the library that cause a dose-dependent increase are reported to be receptors ligands. Two out of the three substances are known to act as ligands for metabotropic glutamate receptor 5 (mGluR5): MPEP being a negative allosteric modulator [36] and VU 0357121 a positive allosteric modulator [37]. The last one, Ciproxifan, is a histamine 3 receptor antagonist [38]. None of these drugs showed any toxicity in the WST-1 assay (Fig 2B). The chemical structures of these three drugs can be found in S1 Fig.

**Table 2:** Comparison between the dose of drugs that led to an increase in PF14:SCO transfection efficacy and the reported IC/EC50 values for the drugs to their respective receptors. The values are calculated from four independent experiments on HeLa pLuc705 and reported as averages ± SEM.

ACTIVATION	EC50 μM ± SEM	Reported Value nM
**VU 0357121**	2,11 ± 0,1	36 (IC50) [37]
**MPEP**	1,16 ± 0,1	33 (EC50) [36]
**Ciproxifan**	0,67 ± 0,2	9,2 (IC50) [38]

A question raised here is why two allosteric modulators with supposedly opposite effects on mGluR5 signaling would have the same effect in this assay. Furthermore, Ciproxifan, VU 0357121 and MPEP’s reported values of IC50 or EC50 for their receptor are, respectively, 73, 59 and 35 folds lower than the measured effect on the transfection efficacy. These contradictory results found in the literature showed us the need for testing our own cell line for the presence of these receptors.

### MPEP, Ciproxifan and VU0357121 do not act through their target receptors

**Fig 3:**
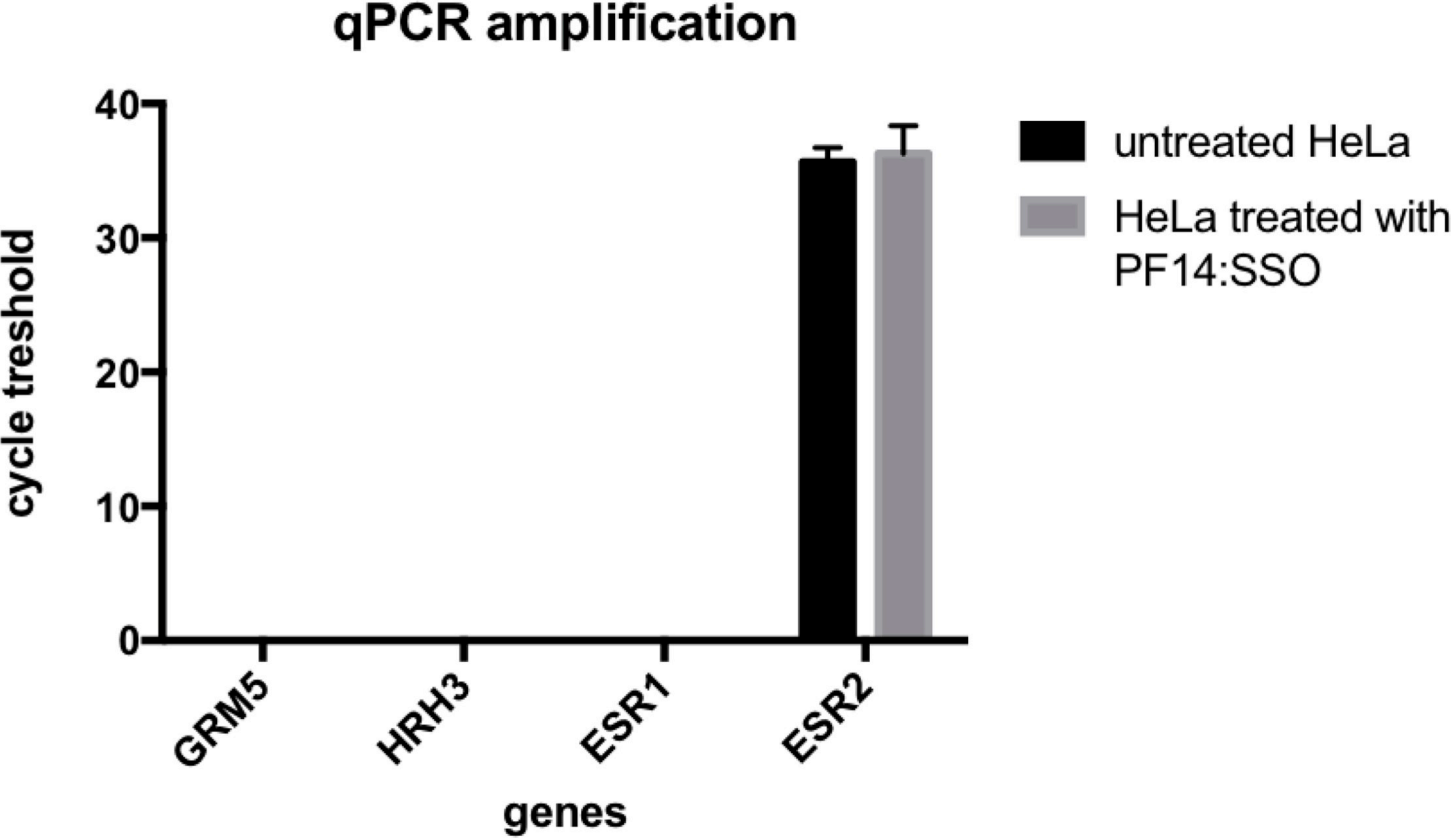
Cycle threshold of qPCR amplification of the genes GRM5, HRH3, ESR1 and ESR2 in HeLa cells treated with the complexes PF14:SCO or not. The values are expressed as a mean + SEM of 3 experiments.

The RNA extracted from the cells with or without treatment was successfully turned into cDNA and then amplified in a RT-qPCR. In our cells the presence of estrogen receptor 2 (ESR2)–coding for estrogen receptor β (ERβ) - was detected but there was no trace of RNA coding for the estrogen receptor 1 (ESR1) (Fig 3). The detection of ESR2 could indicate that a receptor dependent effect of the five estrogen ligands used in this study led to a downstream signal that in return triggered a toxic effect. However, the expression of ESR2 was not changed by our treatment. This suggests that the observed toxicity may not be mediated by the estrogen receptor but might result from another mechanism.

No trace of expression of glutamate metabotropic receptor 5 (GRM5) was found indicating a receptor-independent effect of MPEP and VU0357121. This would explain the similar effect induced by two drugs that have supposedly opposite effect [36,37] as well as the differences between the IC50 and EC50 (Table 1). Furthermore, RNA coding for the histamine receptor H3 (HRH3) was not found either, suggesting that ciproxifan also induced a receptor-independent effect.

It should be noted that the effect reported here was seen with rather high concentrations of drugs compared to their reported values of IC50 and EC50 and it is not unusual for small molecule drugs to have off-target effects at high concentration. Indeed a low affinity for a side target can be compensated by a high concentration. Ciproxifan has already been reported to inhibit the monoamine oxidases A and B (MAO-A and MAO-B) [39]. It has been shown that, while the affinity of ciproxifan for HRH3 varies of about a 100 fold between rats and humans, the IC50 for the inhibition of MAOs for both species were seen in a micromolar range of the drug indicating that the effect on MAOs is not mediated by HRH3 [39]. HeLa cells are known to endogenously express MAO-B [40] but MAOs are involved in the processing of monoamine neurotransmitter, thus it seems difficult to relate this possible inhibition to the observed increased transfection efficacy. In rat striatal neurons treated with MPEP, estradiol failed to induce CREB phosphorylation [41], indicating a connection between a treatment with MPEP and estrogen receptor signaling. This study concluded that the estrogen receptor acts through mGluR5 to induce a MAPK-dependent CREB phosphorylation but it can be noted that the cells were treated with 5 μM of MPEP, a concentration about 140 times higher than its IC50 for mGluR5 [36]. Considering the results of our study it can be hypothesized that MPEP have some off-target effects when used at high concentration and one of these effects could be an interference of the MAPK signaling. Though, such an effect would not explain the increase in the uptake of PF14:SCO complexes as MAPK signaling is mainly involved in transcription factors [42].

Even though this effect of ciproxifan on MAOs at high concentration may not explain the increase in the production of luciferase, it opens this discussion to the concept of dirty drugs. Dirty drugs are a class of drugs that act in an unspecific way, interacting with multiple proteins and signaling pathway. It is not usual to find this term in the research literature; when searching on PubMed in titles and abstracts, “dirty drugs” returns only twenty results. In academic research a preferred term is off-target drugs, nevertheless a definition proposed by Pleyer et al [32] for dirty drugs summarizes the concept as a drug that targets either “several structurally related proteins, a cellular complex composed of several proteins and/or molecules that govern several downstream pathways” [43]. According to this definition, ciproxifan can be considered as a dirty drug or at least as a multi-target drug as it influences at least both HRH3 and MAOs, depending on its concentration. In our study, ciproxifan has an effect on the transfection of a splice correcting oligonucleotide in complex with PepFect14 that is not related to HRH3 and likely unrelated to the inhibition of MAOs. This suggests at least a third signaling pathway influenced by the presence of ciproxifan. MPEP and VU 0357121 induced as well a significant increase of the uptake and transfection efficacy of PF14:SCO without interacting with mGluR5.

### MPEP, VU0357121 and ciproxifan do not significantly change the physico-chemical properties of the nanoparticles

**Fig 4:**
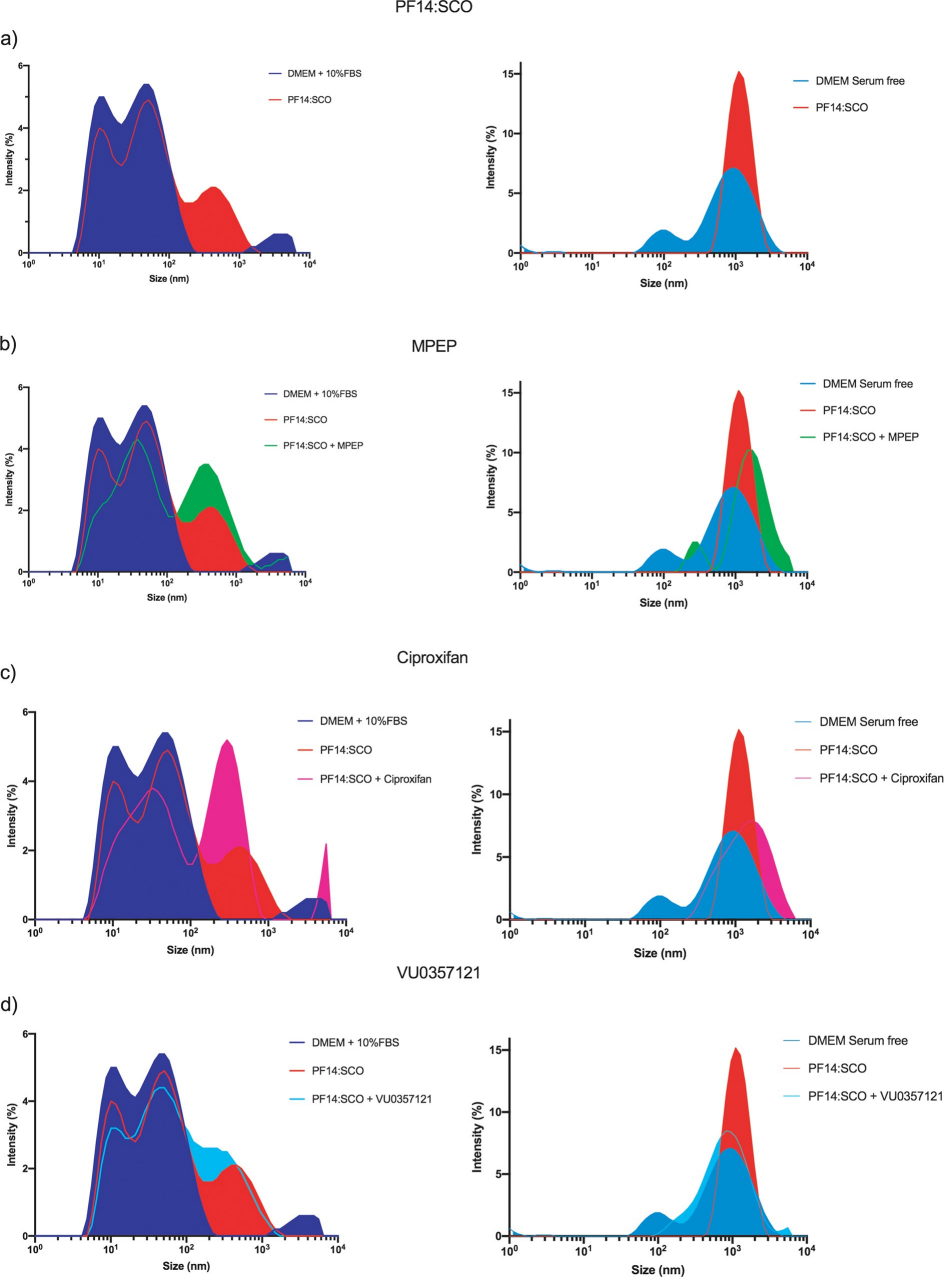
Size distribution of PF14:SCO complexes in presence of the different drugs in DMEM GlutaMAX implemented with 10% FBS or serum free DMEM at 37°C: (a) PF14:SCO (2μM:0.4μM) in media; (b) PF14:SCO (2μM:0.4μM) in presence of MPEP (20μM); (c) PF14:SCO (2μM:0.4μM) in presence of Ciproxifan (20μM); (d) PF14:SCO (2μM:0.4μM) in presence VU0357121 (20μM).

It is not unlikely that our complexes interact with the small drugs. Indeed both of them are already present in the well when we seeded the cell and it is already known that PF14:SCO forms nanoparticles [44]. The short but still existing incubation time of the small drugs with PF14:SCO particles could induce new self-assembled particles changing their physico-chemical properties and thus inducing an enhanced uptake via a different signaling. The PF14:SCO complexes were mixed with the drugs and the hydrodynamic diameter of the particles as well as their ζ-potential were measured. The serum free medium presented two peaks around 100 nm and 1000 nm diameter that were abolished when PF14:SCO was added. Performing the experiment in serum free media allowed the calculation of the Z-average diameter of the particles (Table 3). Even though the average values of the size of the particles showed some differences between the different mixes, the high standard error pointed that they were no significant differences between the samples. In the samples prepared in serum free DMEM, a slight increase of the size could be seen for the complexes prepared with MPEP and ciproxifan whereas a slight decrease of the size was observed for the ones prepared with VU0357121. However no significant differences could be noted. For all readings, particles around 1000 d.nm were found. These results indicated that, in serum free condition, our complexes started to aggregate and were not forming nanoparticles anymore. Nevertheless the actual treatment of the cells was performed in medium containing 10% FBS where the complexes formed nanoparticles between 200 and 400 d.nm (Fig 4, Table 4). In all the readings made in DMEM supplemented with 10% FBS, two particles with diameters around 10 d.nm and 60 d.nm were detected. These two peaks were abolished when using serum-free medium indicating that they were due to the presence of serum in our medium. The serum contains several kinds of proteins including albumin and growth factors that are detected as small particles with a Z-average value of diameter at 21.69 nm. These proteins are present in high number and, therefore, the values of the Z-average diameter in the samples containing PF14:SCO represent mostly the serum with a very high polydispersity index (Table 3). Thus, the diameter of the formed particles was investigated using a single particle size mode on the third peak that appeared when the complexes were added to the serum containing media (Table 4). While the percentage of polydispersity indicated that our particles are not monodisperse, the standard error measured on the diameters showed that all formulations produced particles with a diameter in the range of 10^2^ d.nm. In Fig 4A and Table 4, the diameter of PF14:SCO particles was measured around 300 d.nm in DMEM supplemented with 10% FBS. There was no significant difference in size between the particles in the solutions of PF14:SCO with and without MPEP (Fig 4B, Table 4), though, an increase of intensity at 450 d.nm and a decrease at 10 d.nm could be observed when MPEP was added in the serum containing media. Fig 4C and Table 4 show an increased intensity at 350 d.nm in the sample containing PF14:SCO and Ciproxifan and an overall decrease in the range 5 d.nm—100 d.nm in the sample prepared in FBS containing media. A signal related to a particle bigger than 5000 d.nm is visible in the sample with Ciproxifan but it was out of the detection range of the instrument. A slight difference could be noted between PF14:SCO alone in solution and PF14:SCO together with VU0357121 (Fig 4D, Table 4). The ζ-potential of the particles present in the different mixes prepared in serum containing DMEM or serum free DMEM did not vary significantly (S2 Fig). In serum containing solutions all the values were negatives due to the presence of negatively charged proteins from the FBS. The ζ-potentials were shifted to positive values in serum free DMEM. In none of these two conditions, a significant difference between the formulations could be seen.

**Table 3:** Z-average value and polydispersity index of the particles diameter in serum free DMEM and DMEM supplemented with 10% FBS. The values are presented as mean ± SD.

	Serum-free DMEM		DMEM + 10% FBS	
	Mean size (nm) ± SD	Polydispersity Index	Mean size (nm) ± SD	Polydispersity Index
			21.69 ± 14.61	0.454
**PF14:SCO**	984.8 ± 480.3	0.238	32.99 ± 28,23	0.732
**PF14:SCO + MPEP**	1160 ± 747.2	0.415	35.21 ± 31.45	0.798
**PF14:SCO + Ciproxifan**	1082 ± 598.3	0.306	43.80 ± 35.58	0.660
**PF14:SCO + VU0357121**	623 ± 390.5	0.392	35.45 ± 31,90	0.810

**Table 4:** Diameter of the particles calculated in single particle size mode of the third peaks of the samples prepared in DMEM + 10% FBS.

	DMEM + 10% FBS	
	Mean size (nm) ± SD	% Polydispersity
**PF14:SCO**	295.3 ± 16.,3	47.3
**PF14:SCO + MPEP**	458.7 ± 278.2	49.6
**PF14:SCO + Ciproxifan**	342.0 ± 190.3	52.3
**PF14:SCO + VU0357121**	396.1 ± 239.2	51.9

### MPEP, VU0357121 and ciproxifan induce an endocytosis up-regulation

**Fig 5:**
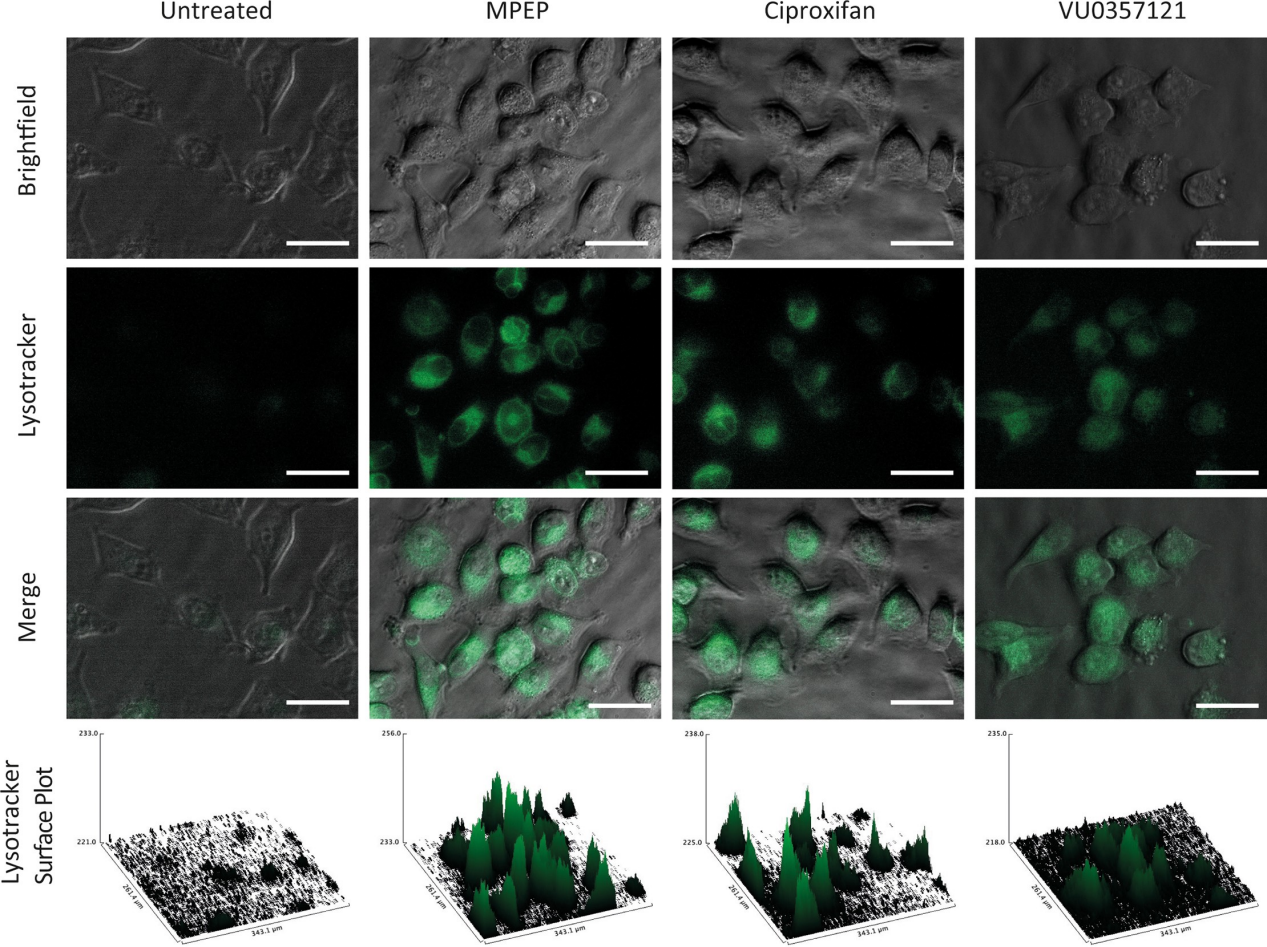
Epi-fluorescence imaging of HeLa pLuc705 cells treated with lysotracker green dye alone or in presence of MPEP (2 μM), Ciproxifan (2 μM) or VU0357121 (2 μM) and their respective surface plots of the green channel. The scale bar represents 50 μM.

**Fig 6:**
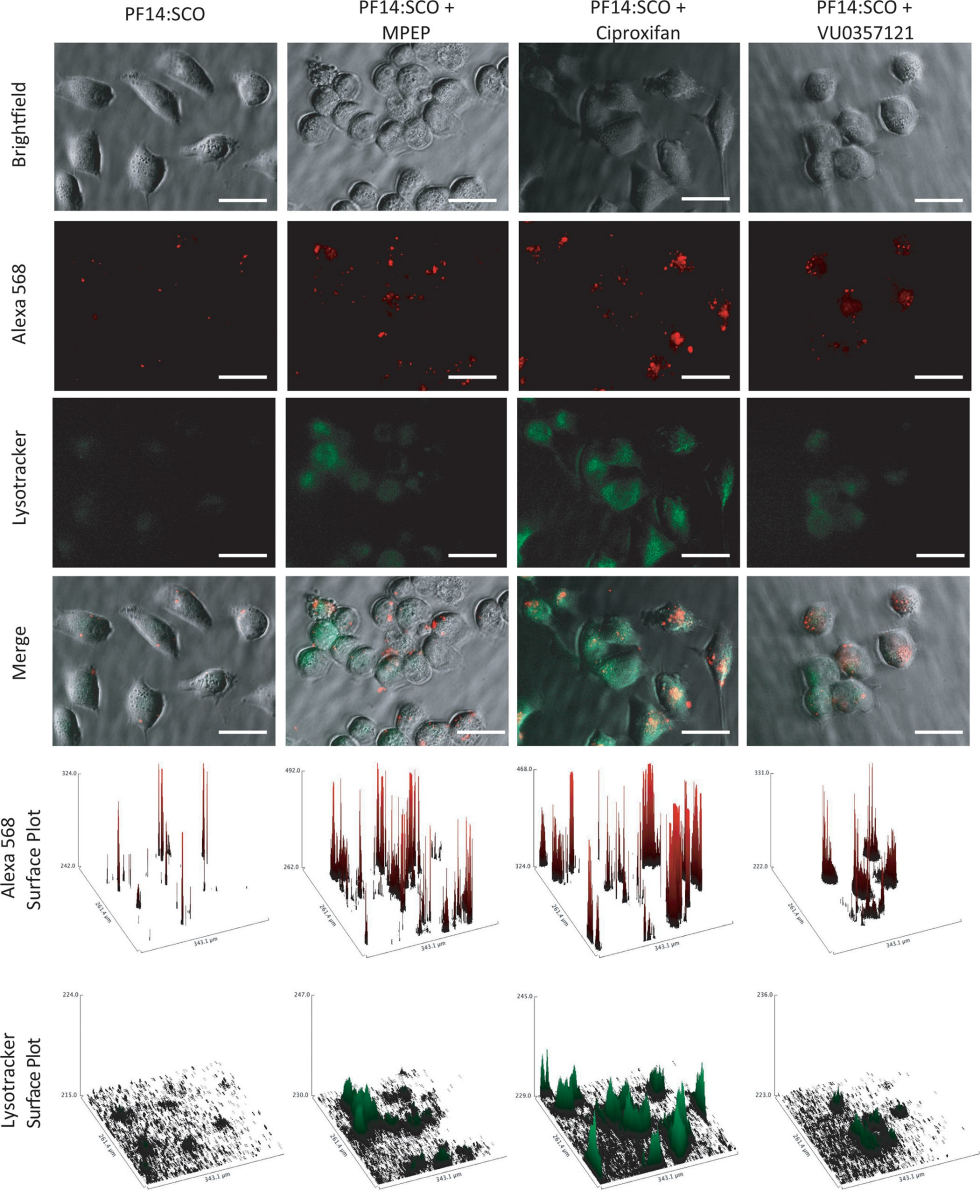
Epi-fluorescence imaging of HeLa pLuc705 cells pre-treated with lysotracker green dye during the uptake of PF14:SCO-Alexa 568 alone, in presence of MPEP (2 μM), Ciproxifan (2 μM) or VU0357121 (2 μM) and their respective surface plots of the red and green channels. The scale bar represents 50 μM.

The cells co-treated with PF14:SCO and the drugs showed an increase in the delivery efficacy (Fig 2). An increase of the delivery efficacy can be due to several mechanisms. In order to determine whether this increase was due to a higher uptake of the complexes or to a better availability of the oligonucleotides inside the cell, a fluorescence microscopy-based assay was performed. HeLa cells were treated with a lysotracker green dye and the different drugs that gave an increase in the transfection efficacy (Fig 5) or pre-treated with the lysotracker and the drugs before addition of the complex formed by PepFect 14 and SCOs coupled to an Alexa 568 fluorophore (Fig 6). The cells were observed with an epi-fluorescence microscope.

In Fig 5, we show the imaging of the cells treated with lysotracker green and the drugs alone. All three drugs induced a clear increase of the lysotracker dye intensity compared to the untreated cells, indicating an effect leading to an increase of the endosomal activity of the cells. This effect could be seen without treating the cells with PF14:SCO and, therefore, is fully due to MPEP, ciproxifan and VU0357121. When the cells were pre-treated with the lysotracker dye and the drugs before co-incubation of the drugs and PF14:SCO-Alexa 568. The same effect could be observed on the endosomal activity monitored by the lysotracker (Fig 6). The Alexa 568 labelling of the SCOs allowed the simultaneous imaging of the oligonucleotides. Clear red fluorescent dots could be seen in most cells, showing that all the treatments had led to the uptake of the PF14:SCO-Alexa complexes to some extent after 1 hour. These spots were due to an accumulation of the fluorescently labelled splice correcting oligonucleotide. These dots can be due to either the accumulation of complexes in intracellular vesicles or on the cell membrane. The surface plots of the alexa 568 dye (Fig 6) allowed a better appreciation of their numbers and spatial densities. In the case of the treatment with PF14:SCO alone only few spots could be seen after 1 hour of treatment. This number drastically increased in the sample that were pre-treated with the drugs followed by the co-incubation with PF14:SCO-Alexa 568. These results clearly indicate an increase of the agglomeration either in endosomes or on the cellular membrane induced by MPEP, ciproxifan and VU0357121. In order to use the same treatment conditions than in the dose dependence assay, HeLa cells were also co-treated with the drugs and PF14:SCO-alexa 568 without pretreatment. The same increase of clear fluorescent red dots was then observed for the cells in presence of MPEP, ciproxifan or VU0357121 (S3 Fig).

**Fig 7:**
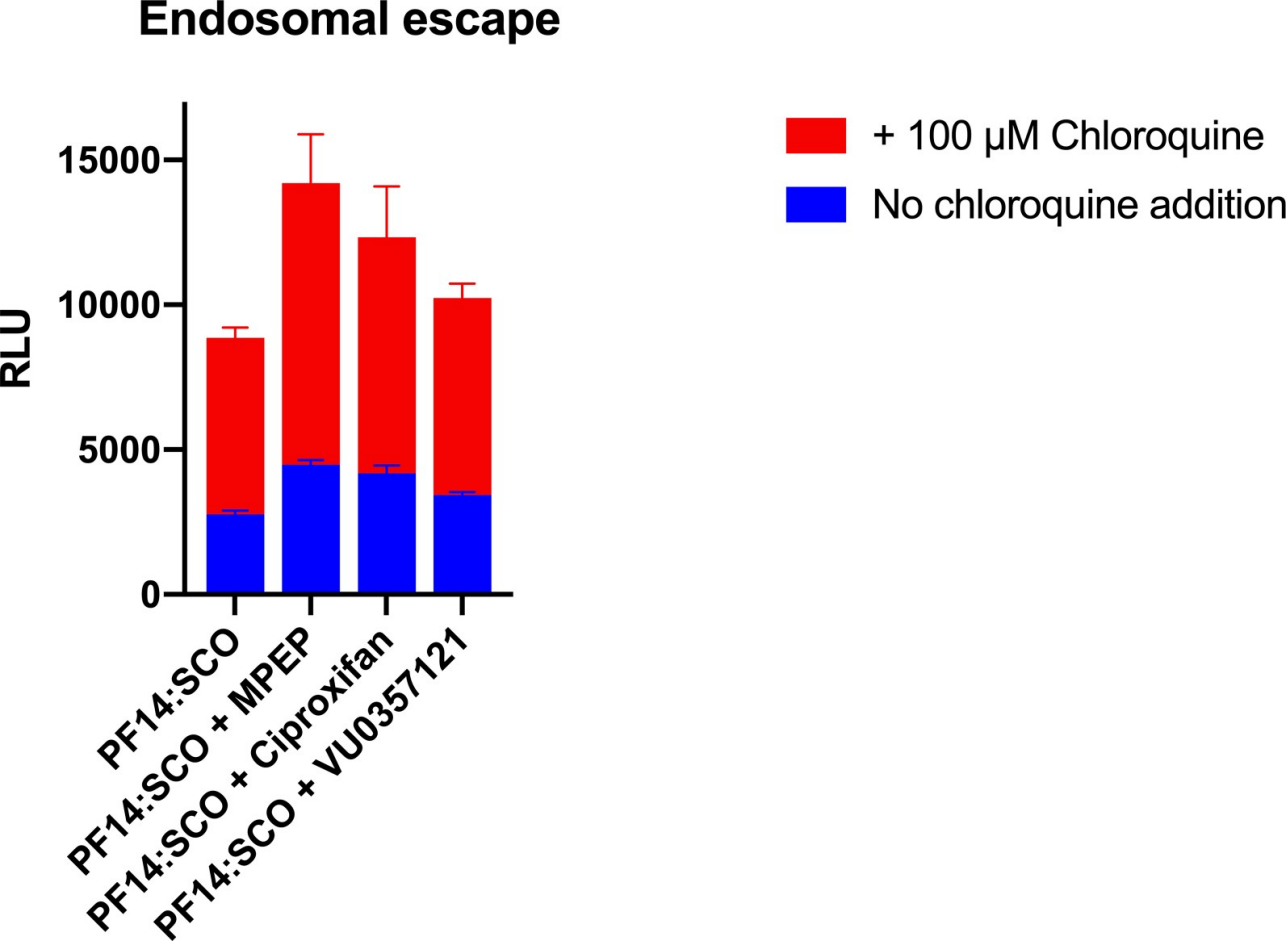
Luminescence induced by the splice correction in the presence or absence of chloroquine for the different treatments (PF14:SCO, PF14:SCO + MPEP (2 μM), PF14:SCO + Ciproxifan (2μM), PF14:SCO + VU0357121 (2 μM)). The experiment was performed on HeLa pLuc705 cells and the results displayed are the mean + SEM of 5 technical replicates.

When the cells were pre-treated with chloroquine, the efficacy of the splice correction showed an even higher increase (Fig 7). Chloroquine is a lysosomotropic agent known to assist the disruption of intracellular vesicles like endosomes. It can diffuse through cell membranes and is taken up in acidic endosomes. There, it acts as a proton sponge that leads to the rupture of the vesicles and the release of their content in the cytoplasm[45,46]. Hence, measuring the effect of chloroquine is an efficient method to detect the endosomal activity of cells. The use of chloroquine gave an increase in the efficacy of PF14:SCO, as already described in Ezzat et al [14], but, more interestingly, chloroquine had an even more important effect on the cells co-treated with PF14:SCO and the small drugs (Fig 7).

Together, Figs 5, 6 and 7 prove that the higher efficacy of the treatment was due to an increased endosomal activity that led to the internalization of more splice correcting oligonucleotide. The increases in the delivery efficacy are then, at least, due to an increase of the uptake of our complexes by an up-regulation of endocytosis. When more complexes are endocytosed, more splice correcting oligonucleotides can be delivered and thus an increase in the production of functional luciferase can be observed. It should be noted that after 1 hour of treatment we observed endosomes but still very little endosomal escape. Neither MPEP, nor ciproxifan, nor VU0357121 assisted a disruption process of the endosomes after 1 hour.

## Conclusions

As of today, the mechanism behind the effect of MPEP, ciproxifan and VU0357121 on the transfection of PF14:SCO is still unclear but as the receptors originally targeted are not expressed by our cells, we can claim that the three drugs present off-targets effects that lead to an increase in the efficacy of PF14:SCO transfection. The study of the particle sizes and surface charges showed that the drugs did not influence significantly the formation of the particles. However the three drugs used alone, in co-treatment or in pre-treatment followed by co-treatment with PF14:SCO induced an increase of endosomal activity, suggesting their involvement in cellular signaling regulating the uptake. We conclude that this three drugs used in μmolar concentrations induce off-target modulation in cell signaling leading to an up-regulation of endosomal processes that led to an increase of PF14:SCO uptake and thus delivery efficacy. These interactions in the signaling could be the result of a yet non-described interaction of the ligands with a cell-surface receptor yielding in a biological effect still not described for these ligands. Another hypothesis is that the high concentration of treatment would allow a direct translocation of the drugs into the cytosol leading to an intracellular interaction on signaling pathways [47].

In summary, we have demonstrated that the use of PepFect 14 in complex with a splice correcting oligonucleotide and three small molecule drugs (VU 0357121, MPEP, ciproxifan) can modulate signaling pathways regulating endocytosis and, as a result of this modulation, led to an increase in the delivery efficacy of PF14:SCO. This finding raises questions about the general transfection mechanism, the involvement of unknown signaling and the specificity of small molecule drugs depending on their concentration.

## Methods

### Cell Culture and Reagents

HeLa pLuc705 cells were grown at 37°C in a humidified incubator with 5% CO2 in Dulbecco’s modified Eagles Medium (DMEM) (Sigma Aldrich, Sweden), supplemented with 10% fetal bovine serum (FBS), 200 μg/ml streptomycin and 200U/ml penicillin (Invitrogen, Sweden). The ligands library was obtained from Selleck chemicals. For the delivery experiments and the luciferin assay buffer, PS-2′-OMe Splice correcting oligonucleotides (5′ -CCU CUU ACC UCA GUU ACA-3′) were purchased from Ribotask (Denmark) and D-Luciferin was purchased from Perkin-Elmer (Sweden). The RNA extraction was performed with RNeasy kit purchased from Qiagen. RevertAid Minus First Strand cDNA Synthesis kit was purchased from ThermoScientific. SsoAdvanced^™^ Universal SYBR^®^ Green Supermix and qPCR primers were bought from Bio-rad (Sweden) (S2 Table). LysoTracker Green DND-26 was purchased from Thermofisher scientific (Sweden). All other reagents, DSMO, MgCO3, MgSO4, Tricine, DTT, CoA, EDTA, WST-1, and ATP were obtained from Sigma-Aldrich (Sweden).

### Peptide synthesis

PepFect 14 (Stearyl- AGYLLGKLLOOLAAAALOOLL-NH2) was synthesized using a microwave-assisted solid phase peptide synthesis instrument (Biotage Alstra+, Biotage AB, Uppsala, Sweden) on H-Rink-Amide-ChemMatrix resin (PCAS Biomatrix, St-Jean-sur-Richelieu (province of Quebec), Canada) using standard Fmoc solid-phase peptide synthesis protocols. Stearic acid (Sigma-Aldrich, Sweden) was coupled to the N-terminus by using HCTU/HOBt [(2-(6-Chloro-1H- benzotriazole-1-yl)-1,1,3,3-tetramethylaminium hexafluorophosphate/6-Chloro-1-Hydroxybenzotriazol] (AGTC Bioproducts, Great Britain & GLSBiochem, China) activation in N-methyl-pyrrolidone (NMP). The coupling steps were performed at 70°C for 5 min, the deprotections were done at room temperature. The peptide was cleaved using 95% TFA, 2.5% water, 2.5% triisopropylsilane for 3 h and precipitated in diethylether. The obtained crude peptide was dried in vacuum, dissolved in water and lyophilized. The peptide was purified via HPLC on a Biobasic C8 column (Thermoscientific, Sweden) using a gradient of acetonitrile / water containing 0.1% TFA. The purity and identity of the purified product were verified by analytical HPLC and by matrix-assisted laser desorption ionization time-of-flight mass spectrometer (MALDI-TOF) (Voyager STE, Applied Biosystems, Sweden). The mass-spectrum was acquired in positive ion linear mode using a-cyano-4-hydroxycinnamic acid as a matrix (Sigma-Aldrich, Sweden) (10 mg/ml, 7:3 acetonitrile: water, 0.1% TFA). After purification peptides where lyophilized. Lyophilized peptides were reconstituted in ultra-pure water (Milli-Q, Merck Millipore) before use. The molarity of the peptide was determined based on dilutions of accurately weighed substances.

### Library screening

The assay was performed as described in Helmfors et al [15], briefly, the PF14:SCO-complexes (2 μM:0.4 μM) were already present in the wells before 7,000 cells were seeded in each well of white clear bottom 96-well microplates. After 24 h the plates were completely emptied of cell medium and frozen at -80°C. After freezing, the plates were brought to room temperature, thus lysing the cells, before the luciferin reagent was added and the luminescence was read. Compared to the assay previously published there was a modification where the ligands from the library were also present in the wells. Since the ligand library was dissolved in DMSO, all cells, including controls, were treated with the same concentrations of DMSO. The dose-dependence experiments were performed the same way. The dose was serially diluted 5:1 in each step starting from 200 μM down to 64 nM. Because of the serial dilution step, the DMSO concentration also decreased 5:1 between each dilution step; the controls were treated the same way and contained the same amount of DMSO. In order to exclude the effects of DMSO, the luminescence readings were normalized in two steps, first to a fold change over the control cells, then to a fold change over the cells treated only with CPP:ON complexes. Due to the number of ligands the assay was performed without replicates. The library screening was repeated three times.

### Toxicity assay

To assess the toxicity of the drugs, a WST-1 assay was done during the same treatments as the ones used for the luciferase assay. 10 μL of WST-1 were added to the medium in each well and incubated for 2h at 37°C. The absorbance of each well was measured at 440 nm over 690 nm. The cell medium with WST-1 was then removed and the cells were lysed for the luciferase assay to be performed. Additionally, the same toxicity assay was performed on HeLa pLuc 705 treated with the drugs alone.

### Luciferase assay

The luciferase assay was performed in the same way as described in Helmfors et al [15]. Briefly, after the lysis of the cells, a solution containing D-luciferin (1mM), ATP (1mM), CoA (25 μM), and DTT (25 mM) with EDTA, MgCO_3_, MgSO_4_ and tricine was added to the lysates and the plate was read in a luminometer. The results were then normalized to untreated cells and the IC/EC 50 values were calculated by fitting the curve in GraphPad Prism for each of four experiments the IC/EC50 values were reported as averages ± SEM from four independent experiments.

### RNA extraction and cDNA synthesis

In a 10 cm Petri dish, the cells were treated with the PF14:SCO-complexes in the same way as previously described. The complexes were already present in the dish when 2.10^6^ HeLa pLuc705 cells were seeded in 9mL of medium. A control was prepared with untreated cells. After 24h the medium was removed and the cells were harvested with the use of trypsin before resuspension in PBS. A pellet was formed by centrifugation and total RNA was extracted using Qiagen RNeasy kit. The two samples were then washed and purified with a Qiagen clean and concentrator kit. RNA purity was verified by absorbance measurements at 230 nm, 260 nm and 280 nm using an Implen P330 nanophotometer. Immediately after, the different RNA samples were turned into cDNA using the RevertAid Minus First Strand cDNA Synthesis kit and the purity of the cDNA samples was verified by absorbance measurements.

### Real time qPCR

The real time qPCR measurements were obtained with primers for metabotropic glutamate receptor 5, histamine receptor H3 and estrogen receptor 1 and 2. Every experiment was done in triplicates. The two cDNA samples concentration were normalized to 50 ng/μL. 100 ng of each cDNA were mixed in a qPCR plate with 10 μL SYBR green probe, 1 μL of the qPCR primers and milliQ water was added to 20μL. The plate was placed in a BioRad iQ5 reader and the amplification cycles were run according to BioRad protocols. Two primers for GAPDH and UBC were used as home keeping gene references.

### Dynamic light Scattering and ζ-potential

The hydrodynamic diameter and the ζ-potential of the particles formed by PF14:SCO with or without small molecule drug was measured using a Zetasizer Nano ZS (Malvern Instruments, United Kingdom). PF14:SCO (20 μM:4 μM) complex was formed in milliQ water at room temperature for 30 minutes. 60 μL of the complex solution was then added to 540 μL of DMEM either serum free or containing 10% of fetal bovine serum to reach a final concentration of 2 μM:400 nM. The 600 μL sample was then transferred to Malvern Disposable Folded Capillary Zeta cells DTS1070 cuvettes and both the hydrodynamic diameter and the ζ-potential were measured. The samples containing the small drug molecules were prepared in the same fashion with the drugs already present in the media before the addition of PF14:SCO. The small molecule drugs final concentration in the cuvettes was 20 μM. All the measurements were performed in duplicates at 37°C.

### Epi-fluorescence microscopy

To prepare the samples, 3.10^5^ HeLa cells were seeded in 3,5 cm Petri dishes with glass-cover bottom and incubated at 37°C overnight. The media was then changed for new media containing 70 nM of Lysotracker green dye either alone or together with each drug (MPEP, ciproxifan and VU0357121) at a concentration of 2 μM for 2 hour. For the samples that were also treated with PepFect14 in complex with splice correcting oligonucleotides labelled with an Alexa 568 fluorophore, the complexes were added to the dishes (2 μM:0.4 μM) after 1h incubation of the lysotracker and the drugs and the cells were incubated for 1 more hour. For all samples, the dishes were then emptied and the cells washed twice with Gibco Opti-MEM cell media and a final addition of 2 mL of Gibco Opti-MEM was let in the dishes. The samples were then used for imaging.

The imaging was performed using a Leica DM/IRBE 2 epi-fluorescence microscope with a 63 × 1.4 NA oil immersion objective for fluorescence imaging, and the images were recorded by a Hamamatsu Orca-ER CCD camera. The system was controlled by the Micro-Manager. Fluorescent images were collected using N3 filter cube (Chroma Technology Corporation, VT, USA) Alexa 568. The surface plots were obtained using Image J software.

### Enhanced endosomal escape

In order to ensure that the increase of the uptake was due to an increase of the endosomal activity of the cells, HeLa pLuc 705 cells were seeded in a 96 well-plate with a density of 7,000 cells per well. The cells were incubated overnight and then were treated with 100 μM chloroquine for 4h. The wells were emptied and fresh media was added to each well. The cells were then treated for one hour with the different drugs (2 μM) before addition of the complexes PF14:SCO (2 μM:0.4 μM). After 24 hour incubation, the wells were emptied and the plate frozen at -80°C overnight before performing the luciferase assay. As a control, in the same plate, cells were not treated with chloroquine.

## Supporting information

S1 Table Small molecule drug library, targets and effects. (PDF)

S2 Table Bio-Rad IDs for the qPCR primers. (PDF)

S1 File A. Normalized toxicity induced by the five estrogen drugs alone on HeLa pLuc 705 cells as measured by the WST-1 assay. Values are calculated from three replicates and are presented as mean + SEM. B. IC50 values of the toxic effect induced by the five estrogen drugs alone on HeLa pLuc 705 cells as measured by the WST-1 assay. Values are calculated from three replicates and reported as mean ± SEM.
(PDF)

S1 Fig Structure of the three drugs that increased the transfection efficacy of PF14:SCO in HeLa pLuc705 cells. (PDF)

S2 Fig Zeta potential of the particles formed in DMEM supplemented with 10% FBS (A) or in serum free DMEM (B). In both case the complex PF14:SCO was at the concentration of 2 μM:0.4 μM and the drugs at 20 μM. The graphs shows the average ± SEM of technical duplicates.
(PDF)

S3 Fig Epi-fluorescence imaging of HeLa pLuc705 cells during the uptake of PF14:SCO-Alexa 568 (a), in presence of MPEP (2 μM) (b), Ciproxifan (2 μM) (c) or VU0357121 (2 μM) (d) and their respective surface plots of the red channel. The scale bar represents 50 μM.
(PDF)
