# Supplementary material for: Effect of small molecule signaling in PepFect14 transfection
Source: PLoS One. 2020 Jan 30;15(1):e0228189. doi: 10.1371/journal.pone.0228189 (PMC6992163; doi:10.1371/journal.pone.0228189)
Supplement: S1 Table — (PDF) [file pone.0228189.s001.pdf]

Drug Name	*CAS Number*	*Target*	*Brief Description*
**VU 0364770**	61350-00-3	GluR	VU 0364770 is a selective positive allosteric mGlu4 with EC50 of 1.1 μM.
**(-)-Huperzine A**	102518-79-6	GluR	(-)-Huperzine A is a potent, highly specific and reversible inhibitor of acetylcholinesterase (AChE) with Ki of 7 nM.
**(+)-Bicuculline**	485-49-4	GABA Receptor	(+)-Bicuculline is a competitive antagonist of GABAA receptors with IC50 of 2 μM.
**(R)-baclofen**	69308-37-8	GABA Receptor	(R)-baclofen is a derivative of gamma-aminobutyric acid primarily used to treat spasticity.
**5-hydroxymethyl tolterodine (PNU 200577)**	207679-81-0	AChR	5-hydroxymethyl tolterodine (PNU 200577) is a new muscarinic receptor antagonist with Kb of 0.84 nM.
**Acebutolol HCl**	34381-68-5	Adrenergic Receptor	Acebutolol is a β-adrenergic receptors antagonist used in the treatment of hypertension, angina pectoris and cardiac arrhythmias.
**Acetylcholine chloride**	60-31-1	AChR	The chemical compound acetylcholine (ACh) is a neurotransmitter in both the peripheral nervous system (PNS) and central nervous system (CNS) in many organisms including humans.
**Acetylcysteine**	616-91-1	AChR	Acetylcysteine is a pharmaceutical drug and nutritional supplement used primarily as a mucolytic agent and in the management of paracetamol (acetaminophen) overdose.
**Aclidinium Bromide**	320345-99-1	AChR	Aclidinium Bromide inhibits human muscarinic AChR M1, M2, M3, M4 and M5 with Ki of 0.1 nM, 0.14 nM, 0.14 nM, 0.21 nM and 0.16 nM, respectively.
**ADL5859 HCl**	850173-95-4	Opioid Receptor	ADL5859 HCl is a δ-opioid receptor agonist with Ki of 0.8 nM.
**Adrenalone HCl**	62-13-5	Adrenergic Receptor	Adrenalone is an adrenergic agonist used as a topical vasoconstrictor and hemostatic, mainly acts on alpha-1 adrenergic receptors.
**ADX-47273**	851881-60-2	GluR	ADX47273 is a potent and specific mGlu5 antagonist with IC50 of 0.17μM.
**Agomelatine**	138112-76-2	5-HT Receptor	Agomelatine is classified as a norepinephrine-dopamine disinhibitor (NDDI) due to its antagonism of the 5-HT2C receptor.
**Alfuzosin HCl (Uroxatral)**	81403-68-1	Adrenergic Receptor	Alfuzosin(Uroxatral) is an alpha1 receptor antagonist used to treat benign prostatic hyperplasia (BPH).
**Allopurinol (Zyloprim)**	315-30-0	OX Receptor	Allopurinol (Zyloprim) is a xanthine oxidase inhibitor with an IC50 of 7.82±0.12 μM.
**Almotriptan malate (Axert)**	181183-52-8	5-HT Receptor	Almotriptan malate (Axert) is a selective 5-hydroxytryptamine1B/1D (5-HT1B/1D) receptor agonist, used for the treatment of Migraine attacks in adults.
**Altrenogest**	850-52-2	Estrogen/progestogen Receptor	Altrenogest is a progestogen structurally related to veterinary steroid trenbolone.
**AM-1241**	444912-48-5	Cannabinoid Receptor	AM-1241 is a selective CB2 agonist with Ki of 3.4 nM.
**AM251**	183232-66-8	Cannabinoid Receptor	AM251 is a cannabinoid 1 receptor antagonist with IC50 of 8.9 μM.
**Amantadine HCl (Symmetrel)**	665-66-7	Dopamine Receptor	Amantadine hydrochloride(Symmetrel) is used to treat or prevent infections of the respiratory tract caused by a certain virus.
**Amfebutamone (Bupropion)**	31677-93-7	Dopamine Receptor	Amfebutamone (Bupropion) is a selective norepinephrine-dopamine reuptake inhibitor with IC50 of 6.5 and 3.4μM for the reuptake of dopamine and norepinephrine, respectively
**AMG-073 HCl (Cinacalcet HCl)**	364782-34-3	CaSR	AMG-073 HCl (Cinacalcet hydrochloride) represents a new class of compounds for the treatment of hyperparathyroidism.
**Amisulpride**	71675-85-9	Dopamine Receptor	Amisulpride is an atypical antipsychotic used to treat psychosis in schizophrenia and episodes of mania in bipolar disorder.
**Amitriptyline HCl**	549-18-8	5-HT Receptor	Amitriptyline inhibits serotonin receptor, norepinephrine receptor, 5-HT4, 5-HT2 and sigma 1 receptor with IC50 of 3.45 nM, 13.3 nM, 7.31 nM, 235 nM and 287 nM, respectively.
**Arecoline**	300-08-3	AChR	Arecoline is a muscarinic acetylcholine receptor agonist.
**Aripiprazole (Abilify)**	129722-12-9	5-HT Receptor	Aripiprazole is a human 5-HT1A receptor partial agonist with a Ki of 4.2 nM.
**ARN-509**	956104-40-8	Adrenergic Receptor	ARN-509 is a selective androgen receptor inhibitor with IC50 of 16 nM.
**Asenapine**	85650-56-2	5-HT Receptor	Asenapine ihibits adrenergic receptor (α1, α2A, α2B, α2C) with Ki of 0.25-1.2 nM and also inhibits 5-HT receptor (1A, 1B, 2A, 2B, 2C, 5A, 6, 7) with Ki of 0.03-4.0 nM.
**Atomoxetine HCl**	82248-59-7	5-HT Receptor	
**Atropine**	5908-99-6	AChR	Atropine sulfate monohydrate is a competitive muscarinic acetylcholine receptor antagonist with an IC50 of 2.5 nM.
**Azasetron HCl (Y-25130)**	123040-16-4	5-HT Receptor	Azasetron HCl is a selective 5-HT3 receptor antagonist with IC50 of 0.33 nM used in the management of nausea and vomiting induced by cancer chemotherapy.
**Azatadine dimaleate**	3978-86-7	Histamine Receptor	Azatadine is an histamine and cholinergic inhibitor with IC50 of 6.5 nM and 10 nM, respectively.
**Azelastine HCl (Astelin)**	79307-93-0	Histamine Receptor	Azelastine hydrochloride (Astelin) is a potent, second-generation, selective, histamine antagonist.
**Bazedoxifene HCl**	198480-56-7	Estrogen/progestogen Receptor	Bazedoxifene HCl is a novel, non-steroidal, indole-based estrogen receptor modulator (SERM) binding to both ERα and ERβ with IC50 of 23 nM and 89 nM.
**Benserazide**	14919-77-8	Dopamine Receptor	Benserazide is a peripherally-acting aromatic L-amino acid decarboxylase (AAAD) or DOPA decarboxylase inhibitor.
**Benztropine mesylate**	132-17-2	Histamine Receptor	Benztropine is a dopamine transporter (DAT) inhibitor with IC50 of 118 nM.
**Bepotastine Besilate**	190786-44-8	Histamine Receptor	Bepotastine is a non-sedating, selective antagonist of histamine 1 (H1) receptor with pIC50 of 5.7.

**Betahistine 2HCl**	5579-84-0	Histamine Receptor	Betahistine is a histamine H3 receptors inhibitor with IC50 of 1.9 μM.
**Betaxolol (Betoptic)**	659-18-7	Adrenergic Receptor	Betaxolol is a selective beta1 adrenergic receptor blocker used in the treatment of hypertension and glaucoma.
**Betaxolol HCl (Betoptic)**	63659-19-8	Adrenergic Receptor	Betaxolol hydrochloride (Betoptic) is a β1 adrenergic receptor blocker with IC50 of 6 μM.
**Bethanechol chloride**	590-63-6	AChR	Bethanechol chloride is a selective muscarinic receptor agonist without any effect on nicotinic receptors.
**Biperiden HCl**	1235-82-1	AChR	Biperiden is an antiparkinsonian agent of the anticholinergic type.
**Bisoprolol**	104344-23-2	Adrenergic Receptor	Bisoprolol is a selective type β1 adrenergic receptor blocker
**Blonanserin (Lonasen)**	132810-10-7	5-HT Receptor	Blonanserin is a novel atypical antipsychotic agent with potent dopamine D2 (Ki, 14.8 nM) and serotonin 5-HT2(Ki, 3.98 nM) receptors antagonist properties.
**BML-190**	2854-32-2	Cannabinoid Receptor	BML-190 is a selective CB2 inverse agonist with Ki of 435 nM.
**BMY 7378**	21102-95-4	5-HT Receptor	BMY 7378 is a multiple inhibitors of α2C-adrenoceptor and α1D-adrenoceptor with pKi of 6.54 and 8.2, respectively.
**Bosentan Hydrate**	157212-55-0	Endothelin Receptor	Bosentan is an endothelin (ET) receptors antagonist for ET-A and ET-B with Ki of 4.7 nM and 95 nM, respectively.
**BRL 54443**	57477-39-1	5-HT Receptor	BRL 54443 is a 5-HT1E and 5-HT1F receptor agonist with pKi of 8.7 and 9.25, respectively.
**BRL-15572**	193611-72-2	5-HT Receptor	BRL-15572 is a 5-HT1D antagonist with pKi of 7.9.
**Brompheniramine**	980-71-2	Histamine Receptor	Brompheniramine is a histamine H1 receptors antagonist.
**Bupivacaine HCl (Marcain)**	18010-40-7	cAMP	Bupivacaine hydrochloride(Marcain) is a more potent cAMP production inhibitor with an IC50 of 2.3 μM
**Canagliflozin**	842133-18-0	SGLT	Canagliflozin is a highly potent and selective SGLT2 inhibitor for CHO-hSGLT2, CHO-rSGLT2 and CHO-mSGLT2 with IC50 of 4.4 nM, 3.7 nM and 2 nM, respectively.
**Carvedilol**	72956-09-3	Adrenergic Receptor	Carvedilol is a non-selective beta blocker/alpha-1 blocker with an IC50 of 3.8 μM for inhibition of LDL oxidation.
**Cetirizine DiHCl**	83881-52-1	Histamine Receptor	Cetirizine Dihydrochloride is an antihistamine.
**Chlorpheniramine Maleate**	113-92-8	Histamine Receptor	Chlorpheniramine (Chlorpheniramine maleate, Chlorphenamine) is an histamine H1 receptor antagonist with IC50 of 12 nM.
**Chlorpromazine (Sonazine)**	69-09-0	Dopamine Receptor	Chlorpromazine (Sonazine) is a dopamine and potassium channel inhibitor with IC50 of 6.1 and 16 μM for nward-rectifying K+ currents and time-independent outward currents
**Chlorprothixene**	113-59-7	Dopamine Receptor	Chlorprothixene has strong binding affinities to dopamine and histamine receptors, such as D1, D2, D3, D5, H1, 5-HT2, 5-HT6 and 5-HT7, with Ki of 18 nM, 2.96 nM, 4.56 nM, 9 nM, 3.75 nM, 9.4 nM, 3 nM and 5.6 nM, respectively.
**Cimetidine (Tagamet)**	51481-61-9	Histamine Receptor	Cimetidine(Tagamet), a histamine congener, competitively inhibits histamine binding to histamine H2 receptors.
**Ciproxifan**	184025-19-2	Histamine Receptor	Ciproxifan is a highly potent and selective H3-receptor antagonist with IC50 of 9.2 nM.
**Cisatracurium besylate (Nimbex)**	96946-42-8	Adrenergic Receptor	Cisatracurium besylate is a nondepolarizing neuromuscular blocking agent, antagonizing the action of acetylcholine by inhibiting neuromuscular transmission.
**Clemastine Fumarate**	14976-57-9	Histamine Receptor	Clemastine Fumarate (Clemastine) is a selective histamine H1 receptor antagonist with IC50 of 3 nM.
**Clomifene citrate (Serophene)**	50-41-9	Estrogen/progestogen Receptor	Clomifene citrate (Serophene) is a selective estrogen receptor modulator.
**Clomipramine HCl (Anafranil)**	17321-77-6	5-HT Receptor	Clomipramine hydrochloride (Anafranil) is a hydrochloride salt of clomipramine which is a serotonin transporter (SERT), norepinephrine transporter (NET) dopamine transporter (DAT) blocker with Ki of 0.14, 54 and 3 nM, respectively.
**Clonidine HCl (Catapres)**	4205-91-8	Adrenergic Receptor	Clonidine hydrochloride(Catapres) is a direct-acting α2 adrenergic agonist with an ED50 of 0.02±0.01 mg/kg.
**Clopidogrel (Plavix)**	120202-66-6	P2 Receptor	Clopidogrel (Plavix) is an oral, thienopyridine class antiplatelet agent.
**Clozapine (Clozaril)**	5786-21-0	5-HT Receptor	Clozapine (Clozaril) is a potent 5-HT1C receptor antagonist with an IC50 of 110 nM for 5-HT-stimulated phosphoinositide hydrolysis.
**CTEP**	871362-31-1	GluR	CTEP is a selective allosteric antagonist of mGlu5 receptor with IC50 of 2.2 nM.
**Cyproheptadine HCl (Periactin)**	969-33-5	Histamine Receptor	Cyproheptadine hydrochloride (Periactin) is a hydrochloride salt form of cyproheptadine which is a histamine receptor antagonist for 5-HT2 receptor with IC50 of 0.6 nM.
**Dapagliflozin**	461432-26-8	SGLT	Dapagliflozin is a potent and selective hSGLT2 inhibitor with EC50 of 1.1 nM.
**Dapoxetine HCl (Priligy)**	129938-20-1	5-HT Receptor	Dapoxetine hydrochloride is a short-acting novel selective serotonin reuptake inhibitor.
**Darifenacin HBr**	133099-07-7	AChR	Darifenacin is a selective M3 muscarinic receptor antagonist with pKi of 8.9.
**Decamethonium Bromide**	541-22-0	AChR	Decamethonium Bromide is a nicotinic AChR partial agonist and neuromuscular blocking agent.
**Desloratadine**	100643-71-8	Histamine Receptor	Desloratadine is a potent antagonist for human histamine H1 receptor with IC50 of 51 nM.
**Desvenlafaxine**	93413-62-8	5-HT Receptor	Desvenlafaxine is a serotonin (5-HT) and norepinephrine (NE) reuptake inhibitor with Ki of 40.2 nM and 558.4 nM, respectively.

**Desvenlafaxine Succinate**	386750-22-7	5-HT Receptor	Desvenlafaxine Succinate is a new serotonin (5-HT) transporter and norepinephrine (NE) transporter reuptake inhibitor with Ki of 40.2 nM and 558.4 nM respectively.
**Detomidine HCl**	90038-01-0	Adrenergic Receptor	Detomidine produce dose-dependent sedative and analgesic effects, mediatated by activation of α2 catecholamine receptors.
**Dexmedetomidine**	113775-47-6	Adrenergic Receptor	Dexmedetomidine is a sedative medication used by intensive care units and anesthetists.
**Dexmedetomidine HCl (Precedex)**	145108-58-3	Adrenergic Receptor	Dexmedetomidine is a highly selective and potent alpha-2 adrenoceptor agonist, which reduces anesthetic requirements for patients by providing significant sedation.
**Dienogest**	65928-58-7	Estrogen/progestogen Receptor	Dienogest is an orally active synthetic progesterone (or progestin).
**Diphemanil Methylsulfate**	62-97-5	AChR	Diphemanil Methylsulfate is a quaternary ammonium anticholinergic, it binds muscarinic acetycholine receptors (mAchR).
**DL-Adrenaline**	329-65-7	Adrenergic Receptor	DL-Adrenaline is a hormone and a neurotransmitter secreted by the medulla of the adrenal glands.
**Domperidone (Motilium)**	57808-66-9	Dopamine Receptor	Domperidone(Motilium) is a dopamine blocker and an antidopaminergic reagent.
**Donepezil HCl (Aricept)**	120011-70-3	AChR	Donepezil is a specific and potent AChE inhibitor for bAChE and hAChE with IC50 of 8.12 nM and 11.6 nM , respectively.
**Dopamine HCl (Inotropin)**	62-31-7	Dopamine Receptor	Dopamine hydrochloride (Inotropin) is a catecholamine neurotransmitter present in a wide variety of animals,And a dopamine D1-5 receptors agonist.
**Doxazosin mesylate**	77883-43-3	Adrenergic Receptor	Doxazosin mesylate is an alpha-1 adrenergic receptor blocker.
**Drospirenone**	67392-87-4	Estrogen/progestogen Receptor	Drospirenone is a synthetic progestin that is an analog to spironolactone.
**Duloxetine HCl (Cymbalta)**	136434-34-9	5-HT Receptor	Duloxetine is a serotonin-norepinephrine reuptake inhibitor with Ki of 4.6 nM, used for treatment of major depressive disorder and generalized anxiety disorder (GAD).
**Empagliflozin (BI10773)**	864070-44-0	SGLT	Empagliflozin (BI-10773) is a potent and selective SGLT2 inhibitor with IC50 of 3.1 nM.
**Enalapril maleate (Vasotec)**	76095-16-4	Opioid Receptor	Enalapril maleate (Vasotec), the active metabolite of enalapril, competes with angiotensin I for binding at the angiotensin-converting enzyme, blocking the conversion of angiotensin I to angiotensin II.
**Epiandrosterone (3β-androsterone)**	481-29-8	Estrogen/progestogen Receptor	Epiandrosterone (3β-androsterone) is a steroid hormone with weak androgenic activity which acts as a L-type Ca2+ channel antagonist.
**Epinephrine bitartrate (Adrenalinium)**	51-42-3	Adrenergic Receptor	Epinephrine bitartrate (D02149, Adrenalinium) is alpha- and beta-adrenergic receptor stimulator.
**Equol**	531-95-3	Estrogen/progestogen Receptor	Equol is an isoflavandiol metabolized from daidzein by bacterial flora in the intestines.
**Escitalopram Oxalate**	219861-08-2	5-HT Receptor	Escitalopram Oxalate is a selective serotonin (5-HT) reuptake inhibitor (SSRI) with Ki of 0.89 nM.
**Estradiol valerate**	979-32-8	Estrogen/progestogen Receptor	Estradiol is a synthetic ester used to treat menopausal symptoms and hormone deficiencies.
**Estriol**	50-27-1	Estrogen/progestogen Receptor	Estriol is an antagonist of the G-protein coupled estrogen receptor in estrogen receptor-negative breast cancer cells.
**Estrone**	53-16-7	Estrogen/progestogen Receptor	Estrone is an estrogenic hormone.
**Ethisterone**	434-03-7	Estrogen/progestogen Receptor	Ethisterone is a progestogen hormone being considered to treat prostate cancer.
**Ethynodiol diacetate**	297-76-7	Estrogen/progestogen Receptor	Ethynodiol diacetate is one of the first synthetic progestogens used in contraceptive pills.
**Etomidate**	33125-97-2	GABA Receptor	Etomidate is a GABAA receptors agonist at GABAA receptors.
**Evista (Raloxifene HCl)**	82640-04-8	Estrogen/progestogen Receptor	Raloxifene is estrogen antagonists, inhibits human cytosolic aldehyde oxidase-catalyzed phthalazine oxidation activity with IC50 of 5.7 nM.
**Famotidine (Pepcid)**	76824-35-6	Histamine Receptor	Famotidine is a histamine H2–receptor antagonist with IC50 of 0.6 mM, commonly used to treat heartburn, GERD, ulcers, and other digestive conditions.
**Fesoterodine fumarate (Toviaz)**	286930-03-8	AChR	Fesoterodine fumarate (Toviaz) is an antimuscarinic agent and is rapidly de-esterified to its active metabolite 5-hydroxymethyl tolterodine that is a muscarinic receptor antagonist.
**Fexofenadine HCl**	153439-40-8	Histamine Receptor	Fexofenadine inhibits histamine H1 receptor with IC50 of 246 nM.
**Fingolimod (FTY720)**	162359-56-0	Bcr-Abl	FTY720 (Fingolimod, Gilenya) is a S1P antagonist with IC50 of 0.033 nM.
**Flavoxate HCl**	3717-88-2	AChR	Flavoxate is a muscarinic AChR antagonist with IC50 of 12.2 μM.
**Flumazenil**	78755-81-4	GABA Receptor	Flumazenil is a benzodiazepine antagonist.
**Fluoxetine HCl**	56296-78-7	5-HT Receptor	Fluoxetine HCl is an antidepressant of the selective serotonin reuptake inhibitor (SSRI)class.
**Fluvoxamine maleate**	61718-82-9	5-HT Receptor	Fluvoxamine maleate is a selective serotonin (5-HT) reuptake inhibitor (SSRI).
**Formoterol hemifumarate**	43229-80-7	Adrenergic Receptor	Formoterol hemifumarate is a potent, selective and long-acting β2-adrenoceptor agonist to β2 and β1 receptors with pKd of 8.12 and 5.58, respectively.
**Forskolin**	66575-29-9	cAMP	Forskolin is a ubiquitous activator of eukaryotic adenylyl cyclase (AC).
**Fulvestrant (Faslodex)**	129453-61-8	Estrogen/progestogen Receptor	Fulvestrant is an estrogen receptor (ER) antagonist with IC50 of 0.094 nM.
**Gabapentin (Neurontin)**	60142-96-3	GABA Receptor	Gabapentin (Neurontin) is a pharmaceutical agent, specifically a GABA analogue.
**Gabapentin HCl**	60142-95-2	GABA Receptor	Gabapentin Hydrochloride is a GABA analogue.

**Galanthamine HBr**	1953-04-4	AChR	Galanthamine (Razadyne, Reminyl) is an AChE inhibitor with IC50 of 14 nM.
**Gallamine triethiodide (Flaxedil)**	65-29-2	AChR	Gallamine triethiodide(Flaxedil) is a cholinergic receptor blocker with an IC50 of 68.0 ± 8.4 μM.
**Gestodene**	60282-87-3	Estrogen/progestogen Receptor	Gestodene is a progestogen hormonal contraceptive.
**Ginkgolide A**	15291-75-5	GABA Receptor	Ginkgolide A is an extract from in Ginkgo biloba and a g-aminobutyric acid (GABA) antagonist with a Ki of 14.5 μM.
**Granisetron HCl**	107007-99-8	5-HT Receptor	Granisetron HCl is a serotonin 5-HT3 receptor antagonist
**Guanabenz (WY-8678) Acetate**	23256-50-0	Adrenergic Receptor	Guanabenz (WY-8678) Acetate is an selective agonist of α2a-adrenergic receptor, α2b-adrenergic receptor and α2c-adrenergic receptor with pEC50 of 8.25, 7.01 and ~5, respectively.
**GW842166X**	666260-75-9	Cannabinoid Receptor	GW842166X is a potent and highly selective agonist of CB2 receptor.
**Hesperetin**	520-33-2	Histamine Receptor	Hesperetin is a bioflavonoid and, to be more specific, a flavanone.
**Hexestrol (Bibenzyl)**	84-16-2	Estrogen/progestogen Receptor	Hexestrol binds to ERα and ERβ with EC50 of 0.07 nM and 0.175 nM, respectively.
**Histamine 2HCl**	56-92-8	Histamine Receptor	Histamine is an organic nitrogen compound, acts on target cells in mammalian brain via stimulation of Histamine 1/2.
**Homatropine Bromide**	51-56-9	AChR	Homatropine Bromide is muscarinic AChR antagonist, inhibits endothelial and smooth muscle muscarinic receptors of WKY-E and SHR-E with IC50 of 162.5 nM and 170.3 nM, respectively.
**Homatropine Methylbromide**	80-49-9	AChR	Homatropine Methylbromide is muscarinic AChR antagonist, inhibits endothelial and smooth muscle muscarinic receptors of WKY-E and SHR-E with IC50 of 162.5 nM and 170.3 nM, respectively.
**Hydroxyzine 2HCl**	2192-20-3	Histamine Receptor	Hydroxyzine is a histamine H1-receptor antagonist, inhibits binding of [3H]pyrilamine/[3H]desloratadine to human histamine H1 receptor with IC50 of 10 nM/19 nM.
**Hyoscyamine (Daturine)**	101-31-5	AChR	Hyoscyamine (Daturine) is an AChR inhibitor with IC50 of 7.5 nM.
**IEM 1754 dihydrobroMide**	162831-31-4	5-HT Receptor	IEM 1754 dihydrobroMide is a selective AMPA/kainate receptor blockers for GluR1 and GluR3 with IC50 of 6 μM.
**Indacaterol Maleate**	753498-25-8	Adrenergic Receptor	Indacaterol is an ultra-long-acting β-adrenoceptor agonist with pKi of 7.36.
**Irsogladine**	57381-26-7	AChR	Irsogladine is an anti-gastric ulcer agent that facilitates gap-junctional intercellular communication through M1 muscarininc acetylcholine receptor binding.
**Isoprenaline HCl**	51-30-9	Adrenergic Receptor	Isoprenaline is a non-selective β-adrenoceptor, inhibits the 3H-inositol phosphate accumulation with IC50 of 0.08 μM.
**Ivabradine HCl (Procoralan)**	148849-67-6	Adrenergic Receptor	Ivabradine, a new If inhibitor with IC 50 of 2.9 μM which acts specifically on the pacemaker activity of the sinoatrial node, is a pure heart rate lowering agent.
**JNJ-7777120**	459168-41-3	Histamine Receptor	JNJ-7777120 is a potent and selective non-imidazole histamine H4 receptor antagonist with Ki of 4.5 nM.
**JTC-801**	244218-51-7	Opioid Receptor	JTC-801 is a selective opioid receptor-like1 (ORL1) receptor antagonist with IC50 of 94 nM.
**Ketanserin (Vulketan Gel)**	74050-98-9	5-HT Receptor	Ketanserin (Vulketan Gel) is specific 5-HT2A serotonin receptor antagonist with a Ki of 2.5 nM for rat and human 5-HT2A.
**Ketotifen fumarate (Zaditor)**	34580-14-8	Histamine Receptor	Ketotifen fumarate (Zaditor) is a fumaric acid salt of ketotifen which is a H1-antihistamine and mast cell stabilizer.
**L-Adrenaline (Epinephrine)**	51-43-4	Adrenergic Receptor	L-Adrenaline (Epinephrine) belongs to a group of the compounds known as catecholamines.
**Lafutidine**	118288-08-7	Histamine Receptor	Lafutidine, a newly developed histamine H(2)-receptor antagonist, inhibits gastric acid secretion.
**LDE225 (NVP-LDE225, Erismodegib)**	956697-53-3	Smoothened	LDE225 (NVP-LDE225) is a smoothened antagonist with IC50 of 1.3 nM (mouse) and 2.5 nM (human), respectively.
**Levosulpiride (Levogastrol)**	23672-07-3	Dopamine Receptor	Levosulpiride is a selective antagonist for D2 dopamine receptors used as an antipsychotic and prokinetic agent.
**Lidocaine (Alphacaine)**	137-58-6	Histamine Receptor	Lidocaine (Alphacaine) is a selective inverse peripheral histamine H1-receptor agonist with an IC50 of >32 μM.
**Loperamide HCl**	34552-83-5	Autophagy	Loperamide is an opioid-receptor agonist with an ED50 of 0.15 mg/kg.
**Loratadine**	79794-75-5	Histamine Receptor	Loratadine is a selective inverse peripheral histamine H1-receptor agonist with an IC50 of >32 μM.
**Lorcaserin HCl**	846589-98-8	5-HT Receptor	Lorcaserin HCl is a selective full agonist of human 5-HT2C receptor with Ki of 15 nM.
**Lurasidone HCl**	367514-88-3	Dopamine Receptor	Lurasidone is an atypical antipsychotic, inhibits Dopamine D2, 5-HT2A, 5-HT7, 5-HT1A and noradrenaline α2C with IC50 of 1.68 nM, 2.03 nM, 0.495 nM, 6.75 nM and 10.8 nM, respectively.
**LY2140023 (LY404039)**	635318-55-7	GluR	LY404039 is a potent agonist of recombinant human mGlu2, mGlu3 receptors and rat neurons expressing native mGlu2/3 receptors with Ki of 149 nM, 92 nM and 88 nM, respectively.
**LY2940680**	1258861-20-9	Hedgehog	LY2940680 binds to the Smo receptor and potently inhibits Hh signaling.
**LY310762**	192927-92-7	5-HT Receptor	LY310762 is a 5-HT1D antagonist with Ki of ~0.2 μM.
**Macitentan**	441798-33-0	Endothelin Receptor	Macitentana (Actelion-1, ACT-064992) is an orally active, non-peptide dual endothelin (ET)A/B receptor antagonist with IC50 of 0.5 nM/391 nM.
**Maprotiline HCl**	10347-81-6	Adrenergic Receptor	Maprotiline hydrochloride (Deprilept, Ludiomil, Psymion) is a selective noradrenalin re-uptake inhibitor and a tetracyclic antidepressant.

**Matrine ((+)-Matrine)**	519-02-8	Opioid Receptor	Matrine((+)-Matrine) is an alkaloid found in plants from the Sophora family. It has a variety of pharmacological effects, including anti-cancer effects, and action as a kappa opioid receptor agonist.
**Medetomidine HCl**	86347-15-1	Adrenergic Receptor	
**Medroxyprogesterone acetate**	71-58-9	Estrogen/progestogen Receptor	Medroxyprogesterone acetate is a progestin, a synthetic variant of the human hormone progesterone and a potent progesterone receptor agonist.
**Memantine HCl (Namenda)**	41100-52-1	AMPA Receptor-kainate Receptor-NMDA Receptor	Memantine hydrochloride (Namenda) is a CYP2B6 and CYP2D6 inhibitor for recombinant CYP2B6 and CYP2D6 with Ki of 0.51 nM and 94.9 μM, respectively.
**Methscopolamine (Pamine)**	155-41-9	AChR	Methscopolamine (Pamine) is a muscarinic acetylcholine receptor blocker.
**Metoprolol tartrate**	392-17-7	Adrenergic Receptor	Metoprolol tartrate is a cardioselective β-adrenergic receptor blocker with IC50 of 42 ng/mL.
**Mianserin HCl**	21535-47-7	Histamine Receptor	Mianserin is a psychoactive agent of the tetracyclic antidepressant.
**Mifepristone (Mifeprex)**	84371-65-3	Estrogen/progestogen Receptor	Mifepristone (Mifeprex, RU-486, RU-38486, Mifegyne) is a remarkably active antagonist of progesterone receptor and glucocorticoid receptor with IC50 of 0.2 nM and 2.6 nM, respectively.
**Mirabegron (YM178)**	223673-61-8	Adrenergic Receptor	Mirabegron is a selective β3-adrenoceptor agonist with EC50 of 22.4 nM.
**Mizolastine (Mizollen)**	108612-45-9	Histamine Receptor	Mizolastine is a histamine H1-receptor antagonist with IC50 of 47 nM used in the treatment of hay fever (seasonal allergic rhinitis), hives and other allergic reactions.
**Mosapride citrate**	112885-42-4	5-HT Receptor	Mosapride is a gastroprokinetic agent that acts as a selective 5HT4 agonist.
**MPEP**	96206-92-7	GluR	MPEP is a selective mGlu5 receptor antagonist with IC50 of 36 nM.
**MRS 2578**	711019-86-2	P2 Receptor	MRS2578 is a potent P2Y6 receptor with IC50 of 37 nM.
**Naftopidil (Flivas)**	57149-07-2	Adrenergic Receptor	Naftopidil (Flivas) is a selective α1-adrenergic receptor antagonist or alpha blocker with a Ki of 58.3 nM.
**Naftopidil DiHCl**	57149-08-3	Adrenergic Receptor	Naftopidil DiHCl is a selective 5-HT1A and α1-adrenergic receptor antagonist with IC50 of 0.1 μM and 0.2 μM, respectively.
**Naloxone HCl**	357-08-4	Opioid Receptor	Naloxone HCl is an opioid inverse agonist drug used to counter the effects of opiate overdose.
**Naltrexone HCl**	16676-29-2	Opioid Receptor	Naltrexone is an opioid receptor antagonist with IC50 of 8 nM used primarily in the management of alcohol dependence and opioid dependence.
**Naphazoline HCl (Naphcon)**	550-99-2	Adrenergic Receptor	Naphazoline hydrochloride (Naphcon) is an ocular vasoconstrictor and imidazoline derivative sympathomimetic amine.
**Naratriptan HCl**	143388-64-1	5-HT Receptor	Naratriptan (Amerge) is a triptan agent that is used for the treatment of migraine headaches.
**Nebivolol (Bystolic)**	152520-56-4	Adrenergic Receptor	Nebivolol selectively inhibits β1-adrenoceptor with IC50 of 0.8 nM.
**Nefiracetam (Translon)**	77191-36-7	GABA Receptor	Nefiracetam (Translon) is a GABAergic, cholinergic, and monoaminergic neuronal systems enhancer for Ro 5-4864-induced convulsions.
**Neostigmine bromide (Prostigmin)**	114-80-7	AChR	Neostigmine bromide(Prostigmin) is a reversible acetylcholinesterase inhibitor.
**Niflumic acid**	4394-00-7	GABA Receptor	Niflumic acid is an inhibitor of cyclooxygenase-2 used for joint and muscular pain.
**Nizatidine**	76963-41-2	Histamine Receptor	Nizatidine is a histamine H2-receptor antagonist with and IC50 of 6.7 nM for AChE.
**Olanzapine (Zyprexa)**	132539-06-1	5-HT Receptor	Olanzapine(Zyprexa) is a high affinity for 5-HT2 serotonin and D2 dopamine receptor antagonist.
**Olopatadine HCl (Opatanol)**	140462-76-6	Histamine Receptor	Olopatadine hydrochloride(Opatanol) is a histamine blocker and mast cell stabilizer with an IC50 of 559 μM for the release of histamine
**Ondansetron (Zofran)**	99614-02-5	5-HT Receptor	Ondansetron (Zofran) is a serotonin 5-HT3 receptor antagonist used mainly as an antiemetic.
**Ondansetron HCl (Zofran)**	99614-01-4	5-HT Receptor	Ondansetron is a serotonin 5-HT3 receptor antagonist.
**Orphenadrine citrate (Norflex)**	4682-36-4	AChR	Orphenadrine citrate is a skeletal muscle relaxant, it acts in the central nervous system to produce its muscle relaxant effects.
**Otenabant (CP-945598) HCl**	686347-12-6	Cannabinoid Receptor	CP-945598 HCl is a potent and selective cannabinoid type 1 receptor antagonist with Ki of 0.7 nM.
**Otilonium Bromide**	26095-59-0	AChR	Otilonium bromide is an antimuscarinic.
**Oxybutynin (Ditropan)**	5633-20-5	AChR	Oxybutynin(Ditropan) is an anticholinergic medication used to relieve urinary and bladder difficulties.
**Oxybutynin chloride**	1508-65-2	AChR	Oxybutynin is an anticholinergic medication used to relieve urinary and bladder difficulties.
**Oxymetazoline HCl**	15-02-08	Adrenergic Receptor	Oxymetazoline hydrochloride is an α1 and α2 adrenergic receptor agonist.
**Paliperidone (Invega)**	144598-75-4	Dopamine Receptor	Paliperidone(Invega) is an atypical antipsychotic.
**Palonosetron HCl**	135729-62-3	5-HT Receptor	Palonosetron HCl is a 5-HT3 antagonist used in the prevention and treatment of chemotherapy-induced nausea and vomiting.
**Pancuronium (Pavulon)**	15500-66-0	AChR	Pancuronium (Pavulon) is a competitive acetylcholine antagonist with an IC50 of 5.5 ¡À 0.5 nM
**Paroxetine HCl**	78246-49-8	5-HT Receptor	Paroxetine is an antidepressant drug of the SSRI type.
**PD 128907 HCl**	112960-16-4	Dopamine Receptor	PD 128907 HCl is a potent and selective dopamine D2/D3 receptors agonist.
**Pemirolast (BMY 26517) potassium**	100299-08-9	Histamine Receptor	Pemirolast potassium (BMY 26517) is a histamine H1 antagonist and mast cell stabilizer that acts as an antiallergic agent.
**Pergolide mesylate**	66104-23-2	Dopamine Receptor	Pergolide mesylate is an antiparkinsonian agent which functions as a dopaminergic agonist.

**PF-5274857**	1373615-35-0	Smoothened	PF-5274857 is a potent and selective Smoothened (Smo) antagonist with IC50 and Ki of 5.8 nM and 4.6 nM, respectively.
**Phenoxybenzamine HCl**	63-92-3	Adrenergic Receptor	Phenoxybenzamine HCl is a non-specific, irreversible alpha antagonist with an IC50 of 550 nM.
**Phentolamine mesilate**	65-28-1	Adrenergic Receptor	Phentolamine mesilate is a nonselective alpha-adrenergic antagonist with IC50 of 0.1 μM.
**Phenylephrine HCl**	61-76-7	Adrenergic Receptor	Phenylephrine hydrochloride is a selective α1-adrenergic receptor agonist.
**Plerixafor (AMD3100)**	110078-46-1	CXCR	Plerixafor is a chemokine receptor antagonist for CXCR4 and CXCL12-mediated chemotaxis with IC50 of 44 nM and 5.7 nM, respectively.
**Plerixafor 8HCl (AMD3100 8HCl)**	155148-31-5	CXCR	Plerixafor 8HCl (DB06809, Mozobil, AMD3100) is the hydrochloride of Plerixafor, a chemokine receptor antagonist for CXCR4 and CXCL12-mediated chemotaxis with IC50 of 44 nM and 5.7 nM, respectively.
**Pramipexole (Mirapex)**	104632-26-0	Dopamine Receptor	Pramipexole (Mirapex) is a partial/full D2S, D2L, D3, D4 receptor agonist with a Ki of 3.9, 2.2, 0.5 and 5.1 nM for D2S, D2L, D3, D4 receptor, respectively.
**Pramipexole 2HCl monohydrate**	191217-81-9	Dopamine Receptor	Pramipexole is a partial/full D2S, D2L, D3, D4, receptor agonist with a Ki of 3.9, 2.2, 0.5, 5.1 nM.
**Prasugrel (Effient)**	150322-43-3	P2 Receptor	A novel platelet inhibitor
**Pregnenolone**	145-13-1	Estrogen/progestogen Receptor	Pregnenolone is an endogenous steroid hormone for inhibition of M1 receptor- and M3 receptor-mediated currents with IC50 of 11.4 ¦ÌM and 6.0 ¦ÌM, respectively.
**Propranolol HCl**	318-98-9	Adrenergic Receptor	Propranolol HCl is a competitive non-selective beta-adrenergic receptors inhibitor with IC50 of 12 nM.
**Prucalopride**	179474-81-8	5-HT Receptor	Prucalopride is a selective, high affinity 5-HT receptor agonistfor 5-HT4a and 5-HT4b with Ki of 2.5 nM and 8 nM, respectively.
**PRX-08066**	866206-55-5	5-HT Receptor	PRX-08066 is a selective 5-HT2B antagonist with IC50 of 3.4 nM.
**Pyridostigmine Bromide (Mestinon)**	101-26-8	AChR	Pyridostigmine Bromide(Mestinon) is a parasympathomimetic and a reversible cholinesterase inhibitor.
**Quetiapine fumarate (Seroquel)**	111974-72-2	Dopamine Receptor	Quetiapine fumarate(Seroquel) is an atypical antipsychotic used in the treatment of schizophrenia, bipolar I mania, bipolar II depression, bipolar I depression.
**Racecadotril (Acetorphan)**	81110-73-8	Opioid Receptor	Racecadotril is a peripherally acting enkephalinase inhibitor with an IC50 of 4.5 μM
**Ramelteon (TAK-375)**	196597-26-9	MT Receptor	Ramelteon (TAK-375) is a novel melatonin receptor agonist for human MT1 and MT2 receptors and chick forebrain melatonin receptors with Ki of 14 pM, 112 pM and 23.1 pM, respectively.
**Ranitidine (Zantac)**	66357-59-3	Histamine Receptor	Ranitidine (Zantac) is a histamine H2-receptor antagonist with IC50 of 3.3 ± 1.4 μM.
**Rimonabant (SR141716)**	168273-06-1	Cannabinoid Receptor	Rimonabant is a selective antagonist of CB1 with IC50 of 13.6 nM and EC50 of 17.3 nM in hCB1 transfected HEK 293 membrane.
**Risperidone (Risperdal)**	106266-06-2	5-HT Receptor	Risperidone(Risperdal) is an atypical antipsychotic used to treat schizophrenia.
**Ritodrine HCl (Yutopar)**	23239-51-2	Adrenergic Receptor	Ritodrine hydrochloride (DU 21220; Miolene; NSC 291565; Pre-Par) is a hydrochloride salt of ritodrine which is a β-2 adrenergic receptor agonist.
**Rivastigmine tartrate (Exelon)**	129101-54-8	AChR	Rivastigmine, a cholinesterase inhibitor with IC50 of 5.5 μM, ues as a parasympathomimetic or cholinergic agent for the treatment of mild to moderate Alzheimer disease.
**Rizatriptan Benzoate (Maxalt)**	145202-66-0	5-HT Receptor	Rizatriptan Benzoate(Maxalt) is a 5-HT1 agonist triptan drug for the treatment of migraine headaches.
**Rocuronium bromide**	119302-91-9	AChR	Rocuronium is an aminosteroid non-depolarizing neuromuscular blocker or muscle relaxant.
**Roxatidine acetate HCl**	93793-83-0	Histamine Receptor	
**RS-127445**	199864-87-3	5-HT Receptor	RS-127445 is a selective 5-HT2B antagonist with pKi of 9.5 and pIC50 of 10.4.
**Rupatadine Fumarate**	182349-12-8	Histamine Receptor	Rupatadine is an inhibitor of PAFR and histamine (H1) receptor with Ki of 550 and 102 nM, respectively.
**Salbutamol sulfate (Albuterol)**	51022-70-9	Adrenergic Receptor	Salbutamol (Albuterol) is a short-acting β2-adrenergic receptor agonist with an IC50 of 8.93 μM.
**SB 271046**	209481-20-9	5-HT Receptor	SB-271046 is a potent, selective and orally active 5-HT6 receptor antagonist with pKi of 8.9.
**SB-269970 HCl**	261901-57-9	5-HT Receptor	SB-269970 HCl is a hydrochloride salt form of SB-269970, which is a 5-HT7 receptor antagonist with pKi of 8.3.
**SB-408124**	288150-92-5	OX Receptor	SB-408124 (Tocris-1963) is a non-peptide antagonist for OX1 with Ki of 57 nM and 27 nM in both whole cell and membrane, respectively.
**SB-742457**	607742-69-8	5-HT Receptor	SB-742457 is a highly selective 5-HT6 receptor antagonist with pKi of 9.63.
**Scopine**	498-45-3	Adrenergic Receptor	Scopine is the metabolite of anisodine, which is a α1-adrenergic receptor agonist and used in the treatment of acute circulatory shock.
**Scopolamine HBr**	114-49-8	AChR	Scopolamine hydrobromide is a competitive muscarinic acetylcholine receptor with an IC50 of 55.3 ¡À 4.3 nM
**Sertraline HCl**	79559-97-0	5-HT Receptor	Sertraline HCl is a 5-HT antagonist with Ki of 13 nM.
**Silodosin (Rapaflo)**	160970-54-7	Adrenergic Receptor	Silodosin(Rapaflo) is an α1-adrenoceptor antagonist with high uroselectivity.
**Sitaxentan sodium (TBC-11251)**	210421-74-2	Endothelin Receptor	Sitaxentan sodium (TBC-11251) is a selective endothelin receptor-A antagonist with IC50 and Ki of 1.4 nM and 0.43 nM, respectively.
**Solifenacin succinate**	242478-38-2	AChR	Solifenacin succinate is a urinary antispasmodic of the antimuscarinic class.
**Sotalol (Betapace)**	959-24-0	Adrenergic Receptor	Sotalol (Betapace) is a non-selective beta blocker and a potassium channel blocker with an IC50 of 43 μM
**Sumatriptan succinate**	103628-48-4	5-HT Receptor	Sumatriptan succinate is a triptan sulfa drug containing a

			sulfonamide group for the treatment of migraine headaches.
**Synephrine HCl**	5985-28-4	Adrenergic Receptor	Synephrine HCl (Oxedrine, p-Synephrine) is a sympathomimetic α-adrenergic receptor (AR) agonist.
**Tamoxifen Citrate (Nolvadex)**	54965-24-1	Autophagy	Tamoxifen Citrate is an antagonist of the estrogen receptor by competitive inhibition of estrogen binding.
**Terbutaline Sulfate**	23031-32-5	Adrenergic Receptor	Terbutaline Sulfate is a selective β2-adrenergic receptor agonist with IC50 of 53 nM.
**Tetrahydrozoline HCl**	522-48-5	Adrenergic Receptor	Tetrahydrozoline HCl is an imidazoline derivative with alpha receptor agonist activity.
**Tianeptine sodium**	30123-17-2	5-HT Receptor	Tianeptine is a selective serotonin reuptake enhancer (SSRE) compound used for treating major depressive episodes.
**Ticagrelor**	274693-27-5	P2 Receptor	Ticagrelor is the first reversibly binding oral P2Y12 receptor antagonist, also inhibits CYP2C9 and 4-hydroxylation with IC50 of 10.5 μM and 8.2 μM respectively.
**Ticlopidine HCl**	53885-35-1	P2 Receptor	Ticlopidine HCl is an P2 receptor inhibitor against ADP-induced platelet aggregation with IC50 of ~2 μM.
**Timolol Maleate**	26921-17-5	Adrenergic Receptor	Timolol Maleate is a non-selective, beta-adrenergic receptor antagonist for β1/β2 with Ki of 1.97 nM/2.0 nM.
**Tiotropium Bromide hydrate**	139404-48-1	AChR	Tiotropium Bromide hydrate is a monohydrate of tiotropium bromide (Spiriva; Tiova; BA 679BR; tiopropium) that is an anticholinergic and bronchodilator and a muscarinic receptor antagonist.
**Tizanidine HCl**	64461-82-1	Adrenergic Receptor	Tizanidine is an α2-adrenergic receptor agonist and inhibits neurotransmitter release from CNS noradrenergic neurons.
**Tolterodine tartrate (Detrol LA)**	124937-52-6	AChR	Tolterodine tartrate (Detrol LA) is a tartrate salt of tolterodine that is a competitive muscarinic receptor antagonist.
**Toremifene Citrate (Fareston, Acapodene)**	89778-27-8	Estrogen/progestogen Receptor	Toremifene Citrate(Fareston, Acapodene) is an oral selective estrogen receptor modulator (SERM) which helps oppose the actions of estrogen in the body.
**Trazodone HCl (Desyrel)**	25332-39-2	5-HT Receptor	Trazodone is an antidepressant belonging to the class of serotonin receptor antagonists and reuptake inhibitors for treatment of anxiety disorders.
**Trimebutine**	39133-31-8	Opioid Receptor	Trimebutine is an agonist of peripheral mu, kappa and delta opiate receptors, used as spasmolytic agent for treatment of both acute and chronic abdominal pain.
**Tripelennamine HCl**	154-69-8	Histamine Receptor	Tripelennamine is a widely used H1 antagonist, inhibiting PhIP glucuronidation with IC50 of 30 μM.
**Tropicamide**	1508-75-4	AChR	Tropicamide is an anticholinergic and a muscarinic receptor subtype M4-preferring antagonist with IC50 of 8.0 nM.
**Tropisetron**	105826-92-4	5-HT Receptor	Tropisetron hydrochloride is a selective 5-HT3 receptor antagonist and α7-nicotinic receptor agonist with an IC50 of 70.1 ± 0.9 nM for 5-HT3 receptor.
**Trospium chloride (Sanctura)**	10405-02-4	AChR	Trospium chloride (Sanctura) is a competitive muscarinic cholinergic receptor antagonist.
**Urapidil HCl**	64887-14-5	5-HT Receptor	Urapidil hydrochloride is a hydrochloride salt form of urapidil which is α1-adrenoceptor antagonist and 5-HT1A receptor agonist with pIC50 of 6.13 and 6.4 respectively.
**Valproic acid sodium salt (Sodium valproate)**	1069-66-5	Autophagy	Valproic acid sodium salt (Sodium valproate) is a HDAC inhibitor with IC50 of 0.4 mM and also inhibits GABA-transaminase or succinic semialdehyde dehydrogenase.
**Varenicline tartrate**	375815-87-5	AChR	Varenicline is a prescription medication used to treat smoking addiction.
**Venlafaxine**	99300-78-4	5-HT Receptor	Venlafaxine is an arylalkanolamine serotonin-norepinephrine reuptake inhibitor (SNRI).
**Vortioxetine (Lu AA21004) hydrobromide**	960203-27-4	5-HT Receptor	Lu-AA21004 is an oral multimodal serotonergic agent, inhibits 5-HT(1A), 5-HT(1B), 5-HT(3A), 5-HT(7) and SERT with IC50 of 15 nM, 33 nM, 3.7 nM, 19 nM and 1.6 nM, respectively.
**VU 0357121**	433967-28-3	GluR	VU0357121 is a novel allosteric modulator of mGlu5 with EC50 of 33 nM.
**VU 0361737**	1161205-04-4	GluR	VU 0361737 is a selective positive allosteric modulator for mGlu4 receptor with EC50 of 240 nM and 110 nM at human and rat receptors, respectively.
**VU 0364439**	1246086-78-1	GluR	
**VUF 10166**	155584-74-0	5-HT Receptor	VUF10166 is a novel, potent and competitive antagonist to 5-HT3A with Ki of 0.04 nM.
**WAY-100635 HCl**	146714-97-8	5-HT Receptor	WAY 100635 is a potent and selective 5-HT agonist with IC50 of 0.95 nM.
**WZ 811**	55778-02-4	CXCR	WZ811 is a highly potent competitive CXCR4 antagonist with EC50 of 0.3 nM.
**Xylazine HCl**	23076-35-9	Adrenergic Receptor	Xylazine HCl is α2 class of adrenergic receptor agonist.
**Zibotentan (ZD4054)**	186497-07-4	Endothelin Receptor	Zibotentan (ZD4054) is a specific Endothelin A (ETA) antagonist with IC50 of 21 nM.
**Zolmitriptan (Zomig)**	139264-17-8	5-HT Receptor	Zolmitriptan(Zomig) is a selective serotonin receptor agonist.

S1 Table. Small molecule drug library, targets and effects.
