# Supplementary material for: Effect of small molecule signaling in PepFect14 transfection
Source: PLoS One. 2020 Jan 30;15(1):e0228189. doi: 10.1371/journal.pone.0228189 (PMC6992163; doi:10.1371/journal.pone.0228189)
Supplement: S2 Table — (PDF) [file pone.0228189.s002.pdf]

Gene name	Primer Biorad UniqueAssayId
**GRM5**	qHsaCID0018089
**HRH3**	qHsaCID0015705
**ESR1**	qHsaCED0033920
**ESR2**	qHsaCED0044944
**UBC**	qHsaCED0023867
**GAPDH**	qHsaCED0038674

S2 Table. Bio-Rad IDs for the qPCR primers.
