# Supplementary material for: Effect of small molecule signaling in PepFect14 transfection
Source: PLoS One. 2020 Jan 30;15(1):e0228189. doi: 10.1371/journal.pone.0228189 (PMC6992163; doi:10.1371/journal.pone.0228189)
Supplement: S1 File — A. Normalized toxicity induced by the five estrogen drugs alone on HeLa pLuc 705 cells as measured by the WST-1 assay. Values are calculated from three replicates and are presented as mean + SEM. B. IC50 values of the toxic effect induced by the five estrogen drugs alone on HeLa pLuc 705 cells as measured by the WST-1 assay. Values are calculated from three replicates and reported as mean ± SEM. (PDF) [file pone.0228189.s003.pdf]

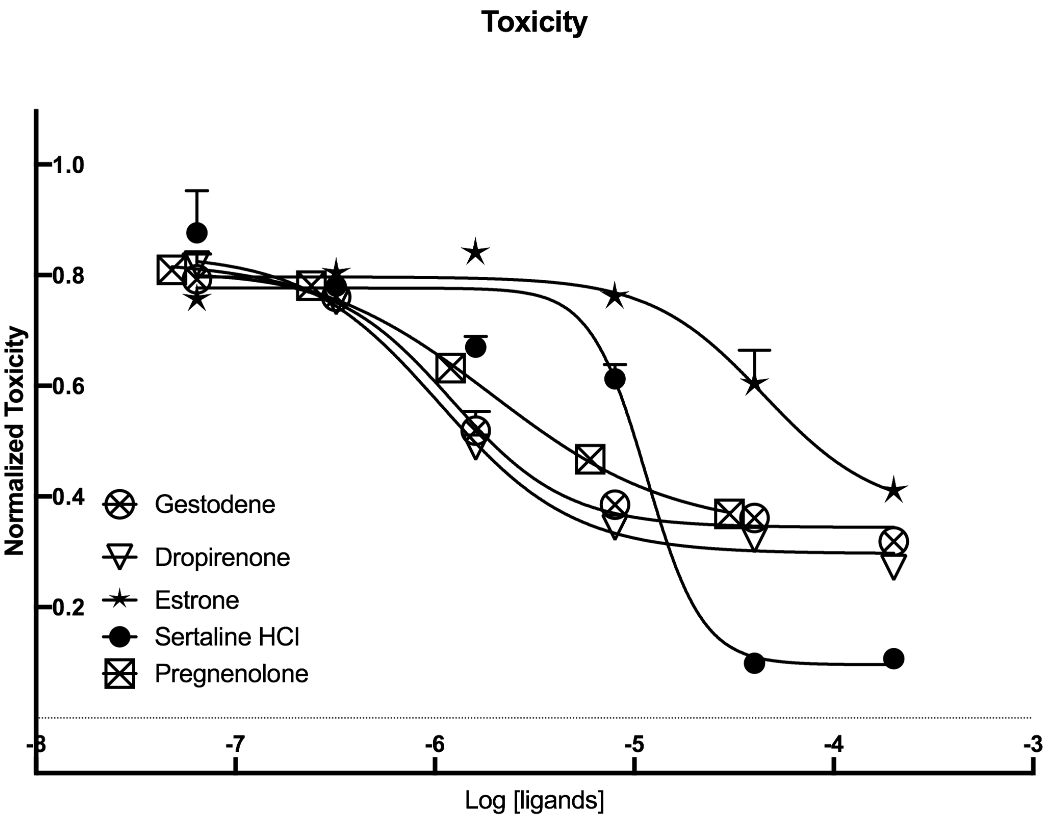

A. Normalized toxicity induced by the five estrogen drugs alone on HeLa pLuc 705 cells as measured by the WST-1 assay. Values are calculated from three replicates and are presented as mean + SEM.

	**Toxicity IC50 µM ± SEM**
**Gestodene**	1,21 ± 1,23
**Drospirenone**	1,06 ± 1,13
**Estrone**	44,51 ± 1,51
**Sertaline HCl**	11,51 ± 1,50
**Pregnenolone**	1,94 ± 1,46

B. IC50 values of the toxic effect induced by the five estrogen drugs alone on HeLa pLuc 705 cells as measured by the WST-1 assay. Values are calculated from three replicates and reported as mean ± SEM.
