# Supplementary material for: Effect of small molecule signaling in PepFect14 transfection
Source: PLoS One. 2020 Jan 30;15(1):e0228189. doi: 10.1371/journal.pone.0228189 (PMC6992163; doi:10.1371/journal.pone.0228189)
Supplement: S1 Fig — (PDF) [file pone.0228189.s004.pdf]

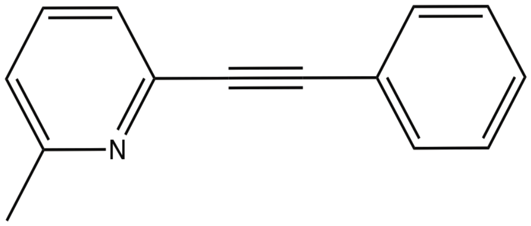

MPEP

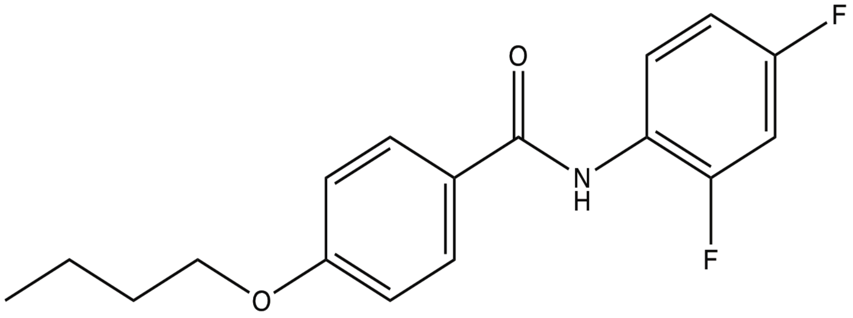

VU0357121

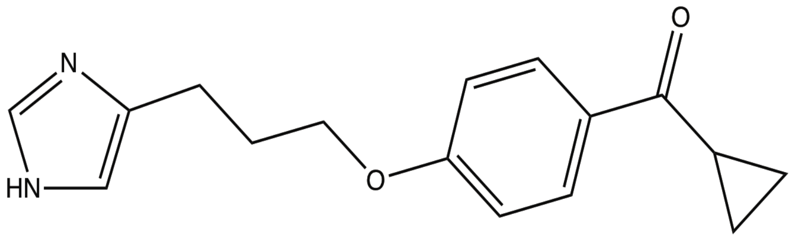

Ciproxifan

S1 Fig. Structure of the three drugs that increased the transfection efficacy of PF14:SCO in HeLa pLuc705 cells.
