# Supplementary material for: Effect of small molecule signaling in PepFect14 transfection
Source: PLoS One. 2020 Jan 30;15(1):e0228189. doi: 10.1371/journal.pone.0228189 (PMC6992163; doi:10.1371/journal.pone.0228189)
Supplement: S2 Fig — Zeta potential of the particles formed in DMEM supplemented with 10% FBS (A) or in serum free DMEM (B). In both case the complex PF14:SCO was at the concentration of 2 μM:0.4 μM and the drugs at 20 μM. The graphs shows the average ± SEM of technical duplicates. (PDF) [file pone.0228189.s005.pdf]

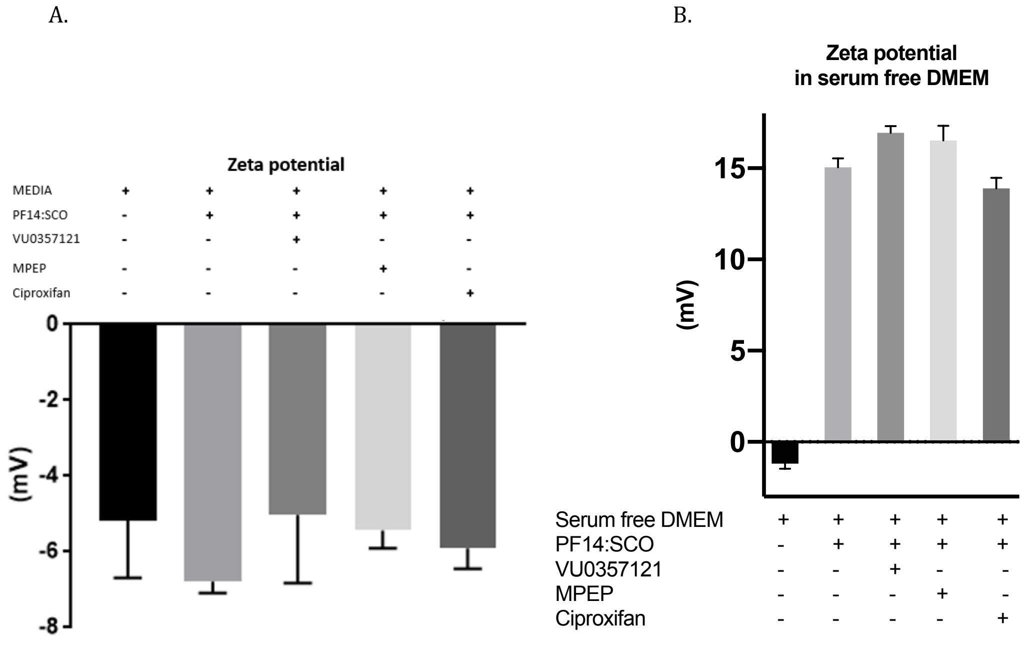

S2 Fig. Zeta potential of the particles formed in DMEM supplemented with 10% FBS (A) or in serum free DMEM (B). In both case the complex PF14:SCO was at the concentration of 2 μM:0.4 μM and the drugs at 20 μM. The graphs shows the average ± SEM of technical duplicates.
