# Supplementary material for: Effect of small molecule signaling in PepFect14 transfection
Source: PLoS One. 2020 Jan 30;15(1):e0228189. doi: 10.1371/journal.pone.0228189 (PMC6992163; doi:10.1371/journal.pone.0228189)
Supplement: S3 Fig — Epi-fluorescence imaging of HeLa pLuc705 cells during the uptake of PF14:SCO-Alexa 568 (a), in presence of MPEP (2 μM) (b), Ciproxifan (2 μM) (c) or VU0357121 (2 μM) (d) and their respective surface plots of the red channel. The scale bar represents 50 μM. (PDF) [file pone.0228189.s006.pdf]

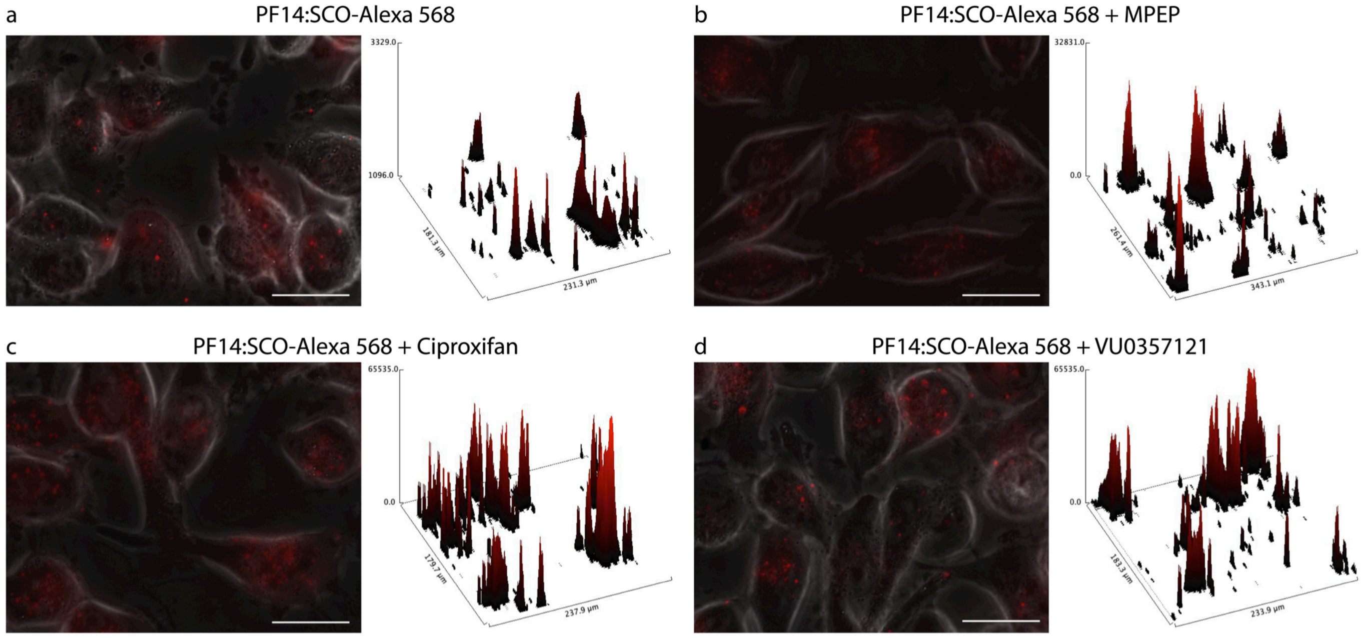

S3 Fig. Epi-fluorescence imaging of HeLa pLuc705 cells during the uptake of PF14:SCO-Alexa 568 (a), in presence of MPEP (2 μM) (b), Ciproxifan (2 μM) (c) or VU0357121 (2 μM) (d) and their respective surface plots of the red channel. The scale bar represents 50 μM.
